# The green solvent: a critical perspective

**DOI:** 10.1007/s10098-021-02188-8

**Published:** 2021-09-30

**Authors:** Neil Winterton

**Affiliations:** Department of Chemistry, University of Liverpool, Liverpool, L69 7ZD UK

**Keywords:** Solvents, Research & Development, Waste minimisation, Sustainable development

## Abstract

Solvents are important in most industrial and domestic applications. The impact of solvent losses and emissions drives efforts to minimise them or to avoid them completely. Since the 1990s, this has become a major focus of green chemistry, giving rise to the idea of the ‘green’ solvent. This concept has generated a substantial chemical literature and has led to the development of so-called neoteric solvents. A critical overview of published material establishes that few new materials have yet found widespread use as solvents. The search for less-impacting solvents is inefficient if carried out without due regard, even at the research stage, to the particular circumstances under which solvents are to be used on the industrial scale. Wider sustainability questions, particularly the use of non-fossil sources of organic carbon in solvent manufacture, are more important than intrinsic ‘greenness’. While solvency is universal, a universal solvent, an alkahest, is an unattainable ideal.

**Supplementary Information:**

The online version contains supplementary material available at 10.1007/s10098-021-02188-8.

## Introduction

**Fig. 1:**
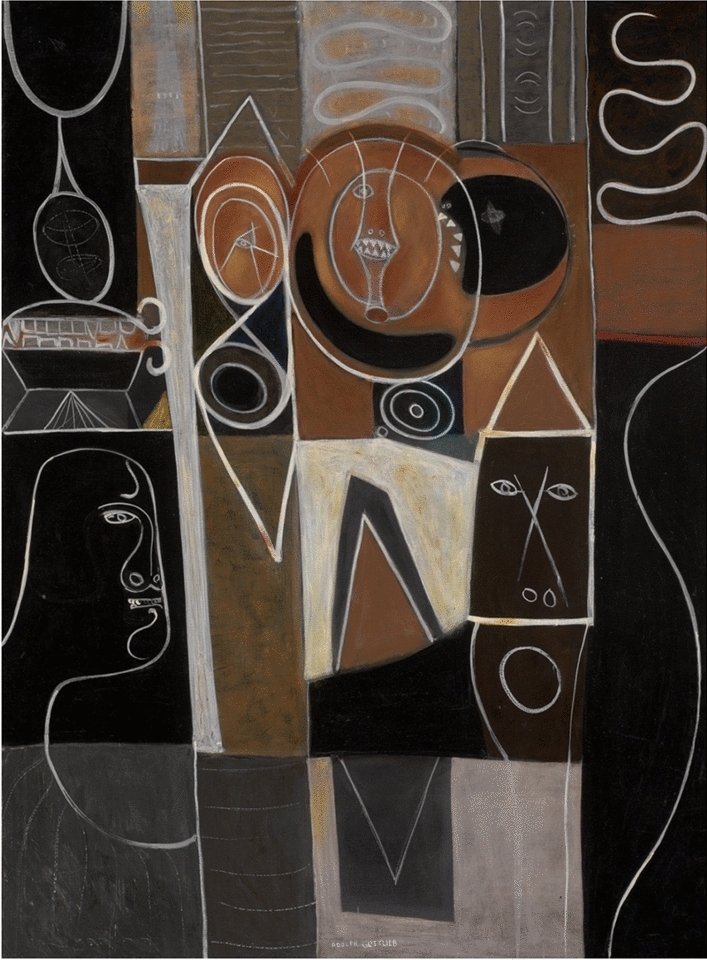
The Alkahest of Paracelsus by Adolph Gottlieb (1903–1974), Museum of Fine Arts, Boston ©Adolph and Esther Gottlieb Foundation (Alkahest of Paracelsus, 1945 by Adolph Gottlieb (American) 1903–1974. Oil and egg tempera on canvas 152.4 × 111.76 cm (60 × 44 in.). Museum of Fine Arts, Boston, Tompkins Collection—Arthur Gordon Tompkins Fund 1973.599.). (Photograph © Museum of Fine Arts, Boston.) (http://www.mfa.org/collections/object/alkahest-of-paracelsus-34184)

The American artist, Adolph Gottlieb, painted ‘*The Alkahest of Paracelsus*’ (Fig. 1) in 1945 in the aftermath of the Second World War. The painting is described as ‘*a pictorial alkahest, a visual solvent intended to strip away illusions and expose the truth of a civilization tottering on the edge of self-destruction*’. Such are the challenges of sustainability, exacerbated by those of the COVID-19 pandemic, that we might conclude that Gottlieb’s painting conveys a similar grim message seventy five years later.

This perspective considers the idea and the reality of the ‘green’ solvent and explores the search by green chemists for a modern ‘alkahest’. It reviews the characteristics of the ideal solvent, examines the complexities of solvent selection and solvent replacement^1^ and assesses the likelihood that one can be identified.

Sections “Solvents and solvency” and “Solvents use, waste and pollution” provide a brief introduction to solvents, their uses and their impact. Section “Solvent replacements” introduces solvent replacement methods for the reduction of such impact, followed by “Solvents and sustainability” section, which summarises efforts to identify new solvents which are more sustainable, within which the idea of the ‘green’ solvent is critically considered.

## Solvents and solvency

Solvency, solvents and solutions are part of everyday life, from making a cup of coffee (and putting sweetener in it and washing the cup afterwards) to washing one’s hair in the shower, from applying and removing nail varnish to rinsing an apple under the tap before eating it, from applying a coat of paint to taking a soluble pain killer for a headache. But what is a solution? What is a solvent or a solute? What is the difference between a solution and a suspension, or a dispersion, a gel or a slurry?

The chemistry of solutions and solvents, therefore, is of interest in its own right (Reichardt and Welton 2010), being of wide chemical relevance, encompassing phase behaviour and the nature of solids, liquids and gases and the properties of mixtures of mainly liquids and solids. Solvation encompasses all types of inter-molecular interaction, such as van der Waals bonding, ionic/dipolar interactions, hydrogen bonding and charge transfer. Solvents used as media for chemical reactions have a profound effect on both the thermodynamics and kinetics of physical and chemical processes (Buncel et al. 2003).

Precisely which solvent will dissolve which solute and which solute will dissolve in which solvent (and why) are determined by a delicate balance of a range of factors that include the differing nature and relative strengths of inter-molecular forces in the pure solute and solvent and interactions between molecules of the solute and solvent (coupled with entropic factors governing the overall process of solute + solvent → solution). The simplest and oldest rule of thumb is that ‘*like dissolves like*’. A polar material, like common salt, sodium chloride, is more likely to dissolve in a polar solvent, like water, than in a non-polar solvent, like hexane. This empirical idea has been refined and extended (Barton 1991) (and will be discussed further in “Solvent selection and design: empirical database and computational methods” section) so that certain molecule-specific properties (such as polarisability, dipolarity, hydrogen-bond donor and acceptor ability) can be put into numerical form as ‘solubility parameters’ (Hansen 1969) or, latterly, as ‘solvatochromic parameters’ (Marcus 1993; Kamlet et al. 1986) for both solutes and solvents. Tables of such data are now available to guide the screening of the large number of possible solvents for those most likely (singly or in combination) to dissolve a solute of choice. Recent work, also discussed in “Solvent selection and design: empirical database and computational methods” section, has sought to incorporate toxicological and environmental characteristics into such screening.

Everyday use of solvents, such as in washing dishes or in cleaning the floor, involves the dissolution (or removal in some other way) of a complex mixture of materials, some adhering to the surface to be cleaned with varying degrees of tenacity. It is not surprising, therefore, that products designed for this purpose are formulations containing several components (some liquid, some solid) each designed to fulfil a particular additional function, such as wetting, penetration or dispersion (or even making the product smell nice), and not simply dissolution. Is the ‘solvent’ in this context the entire composition, or one (or all) of the liquid components?

How should one address the overall acceptability of such diverse products? Should they primarily be judged on the basis of the efficacy of the cleaning process (and how would one judge that?); or the cost-effectiveness of the product in bringing about the desired effect. Would one be prepared to pay more for an ‘excellent’ cleaning outcome compared with an ‘acceptable’ outcome? Or, should it be based on the environmental impact of the product, and would one be prepared to accept an inferior cleaning outcome if the product was (however judged) more environmentally benign?

Would these criteria be the same or different if one were to consider different types of solvent, such as one used as a paint stripper or nail-varnish remover or a shampoo rather than a floor cleaner? And, would the priority given to one criterion over another also be different, depending on how it was used?,^2^^,^^3^

## Solvents use, waste and pollution

The environmental and other consequences that arise from the large-scale use of solvents highlight the need to reconcile their usefulness with the minimisation of their impact. This has been a long-standing pre-occupation that has seen compounds used as solvents, such as *benzene*,^4^*carbon disulfide* and *carbon tetrachloride,* first used and then substituted as new substances became available which were more efficacious or less hazardous. Other solvents now also used much less in industry include *diethyl ether* (because of its flammability and tendency to form peroxides), *di-*iso*-propyl ether* (peroxide formation), *hexane* (neurotoxicity), *nitromethane* (explosion hazard) and *ethylene glycol dimethyl ether* (teratogenicity).

**Fig. 2:**
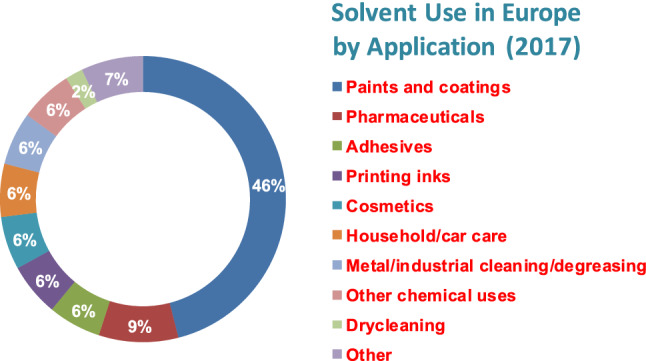
Solvents used in a variety of industrial applications in Europe in 2017 (http://www.esig.org)

Solvents are ubiquitous and have a wide range of industrial uses (Table 1). It is estimated (http://www.ihs.com/products/chemical/planning/special-reports/global-solvents.aspx) that *ca* 28 million tons of solvents are used annually. About 5 million tons are used by the European solvents industry alone (Fig. 2). It is likely that similar quantities are emitted to the environment. European emissions inventories (http://www.esig.org/wp-content/uploads/2020/06/ESIG-technical-paper-solvent-VOC-emissons-2018-final-corrected.pdf.) report 2–3 million tons of VOCs emitted per year during the period 2008–2018.

**Table 1:** A selection of the major uses for solvents

Solvent extraction	Product extraction (fermentation; phytochemical)
	Hydrometallurgy
	Waste water treatment
Cleaning	Metal degreasing
	Dry cleaning
	Domestic cleaning
Formulations	Dispersant
	Lubricant
	Surfactant
	Adhesives
	Viscosity modifier
	Diluent
Coatings	Paints
	Varnishes
Chemicals production	Reaction medium
	Product purification
	Chromatographic solvent

The wide range of solvents used in industry can be seen from early compilations (Flick 1998; Cheremisinoff 2003) which list > 1200 compounds from most compound types.

Waste in all its forms, its sources (domestic, institutional and industrial), impact and methods of ultimate disposal have long been a matter of public concern. There has, over the last 40–50 years, been increasing emphasis (through legislation, regulation, public pressure and competition) on the amount of waste produced, particularly in the chemical industry. A waste minimisation hierarchy (http://en.wikipedia.org/wiki/Waste_hierarchy), developed in the 1970s for the chemical process industry, was devised to encourage purposeful reduction in chemical waste, including solvents.

The terms ‘waste’ and ‘pollution’ tend to be used interchangeably, though in the case of chemicals production it is sometimes useful to make a distinction between them: ‘*waste*’ includes material, such as a stoichiometric **co**-product, which is *inevitably* produced in a process, as well as other **by**-product or processing material that has no commercial value. The safe disposal of such materials incurs costs; ‘*pollution*’, on the other hand, has come to be thought of as any product (waste or otherwise) which finds its way into the environment and may cause harm (however, defined). Solvents can fall into either category.

The four key sectors of the chemicals industry—oil refining, bulk or commodity chemicals, fine or effect chemicals and pharmaceuticals—differ in the proportion of by-product and waste formed in product manufacture (Sheldon 1992, 2017). This is highest for pharmaceuticals much of whose production uses multi-step liquid-phase batch operations. A multiplicity of solvents is used in the preparation, isolation and purification of intermediates at various stages of the production of a single active pharmaceutical ingredient (API).^5^ As much as 80% of life cycle process waste (excluding water) from the manufacture of APIs may arise from the use of solvents (Jiménez-González et al. 2004).

Applying the waste minimisation hierarchy to the use of solvents (and other by-products) in chemicals manufacture seeks to prioritise, first, *avoidance* (the development of ‘solvent-free’ processes (Kerton and Marriott 2013f; Tanaka and Toda 2000) or the total elimination of the source of a solvent emission), then *minimisation* [via solvent *reduction*, *recovery, reuse* and *recycle*) and finally, safe *disposal* (conversion to less harmful materials or complete destruction (with energy recovery, if appropriate)]. Each of these aspects continues to be the focus of research.

Note, however, solvent-containing formulated products generally emit volatile components during or after use. The highly distributed use of many such products, particularly those for use domestically, makes them very difficult, if not impossible, to contain or recover. Approaches to minimise impact are therefore different from those used in chemicals manufacture, being more reliant on solvent substitution or minimisation.

## Solvent replacements

Reduced solvent impact can come about by avoidance of emissions and by replacing solvents of concern. Complete avoidance, both of emissions or solvents, usually requires major innovative changes in reaction engineering and process technology. Replacements for currently used solvents can be found either by (i) re-purposing existing chemical substances^6^ or (ii) by discovering some wholly new materials. Route (i) is generally taken by industry, though both industry and academia are involved in developing methods which streamline and refine the search for alternatives to industrial solvents. Route (ii) is generally, though not universally, the path taken in academia, often with financial support from, or in collaboration with, industry.

To the need to limit harmful or potentially harmful chemicals emissions must now be added the requirements of sustainability. As a consequence, a major effort has grown up both in industry and academia seeking new solvents from more renewable feedstocks, such as biomass, and the development of process technologies making them. This is discussed in “Biosolvents or bio-derived solvents” and “Solvents from other renewable sources” sections.

Historically, it was the case that the exploratory and discovery stages in synthesis^7^ and new product development, particularly in fine chemicals and pharmaceuticals, limited its initial consideration of solvent use and impact to matters of occupational health in the laboratory environment. Techno-commercially more suitable alternatives could be considered during the development phase, that is, during the transition between the research laboratory and the market place.

Increased regulatory, customer and user expectation, added to competitive pressure, as well as to reduce development costs and time to market, the question of the suitability and acceptability of solvents (indeed, any volatile compound) is now more likely to be addressed at the discovery stage. Indeed, such factors are increasingly built in as laboratory programmes are formulated, often taking account of the concepts of sustainable technologies (Winterton 2021a, b).

The dilemmas involved can be illustrated with an example. Improving the conversion efficiency of solar cells for the direct conversion of sunlight into electricity is a major fundamental and technological challenge. Prospects have been boosted recently by impressive research results from studies of inorganic–organic hybrid perovskites. Key has been the fabrication of bilayer structures. In one example (Jeon et al. 2014), a five-step spin-coating process used a solution of complex lead^8^ salts in a mixture of *γ-butyrolactone* and *dimethylsulfoxide* (DMSO) (7:3 by volume) with *toluene* as the anti-solvent (Kang et al. 2020) to effect deposition. The development challenge must reconcile the potential benefits of low-cost low-carbon solar energy with the environmental and toxicological impact of materials (including the solvents) used to deliver it. Similar solvent-related studies (Liu and Xu 2020) have been reported in the processing and fabrication of organic photovoltaics, battery electrodes (Goren et al. 2016) and electronic materials more generally (Li et al. 2020). A 2019 review of organic photovoltaics (Ma et al. 2019) reveals the challenges of changing solvent while retaining key technical and techno-commercial characteristics. However, while these replacement solvents (*xylenes*, *tetrahydrofuran*, *DMSO*, *chlorobenzene*, thiophene, *diphenyl ether*) are frequently described as green, little evidence is cited to support these descriptions. Indeed, one paper (Zhang et al. 2016) uses the term ‘green’ as a relative term, pointing out that ‘*the green solvent discussed here is a concept relative to the traditional halogenated solvents and not the well-defined green solvents of green chemistry*’. Materials newly applied in these applications include *cyclopentyl methyl ether*, N,N′*-dimethylpropyleneurea*, *diethyl succinate*, *isobutyl acetate*, *dimethyl carbonate*, t*-amyl methyl ether* and 2-methylanisole (Lee et al. 2017). Some of these are also targets in the search for solvents from biomass.

### Solvent guides from the pharmaceutical industry

Manufacture of pharmaceuticals involves the production, to exacting standards, of formulated products using complex reaction sequences, often reliant on a specific solvent or set of solvents as reaction medium. Intermediates frequently require isolation and purification between stages, often employing different solvents. This accounts, in large part, for the high E-factor [the ratio (kg/kg) of waste to useful product for a process (Sheldon 1992, 2017)] for the pharmaceuticals sector. The impact and costs of losses and emissions drive efforts to limit them as well as the search for alternatives with reduced impact.

The most extensively and widely used solvents are listed in a series of Solvent Selection Guides produced by manufacturers of pharmaceuticals, responding to the need to limit emissions of VOCs and to replace solvents of concern in chemicals production. These Guides were summarised in 2014 (Prat et al. 2014) and comprehensively reviewed in 2016 (Byrne et al. 2016). The 154 solvents in widespread use in pharmaceuticals production listed in the Glaxo Smith Kline, Pfizer, Sanofi, Astra-Zeneca guides and from an industry consortium, GCI Pharmaceuticals Roundtable, are assembled in Table S1 (Online Resource 1).^9^

The solvents listed come from a range of solvent types, classified according to their composition (aliphatic or aromatic hydrocarbon, oxygenated, halogenated), the presence of one or more functional groups (alcohol, ester, ether, ketone, carboxylic acid, amine, sulfoxide, sulfone, phosphoramide) or their molecular, physical or chemical characteristics relevant to solvent property or behaviour (such as non-polar, dipolar, polar aprotic, polar, acidic, basic, hydrogen-bond donor or acceptor) and various assessments of safety, health and environmental impact. All those listed in Table S1 are long-known chemical species. Indeed, according to data accessed via their CAS Registry Number (RN)^10^ most have been known for more than 100 years. This is hardly surprising as the compounds listed in the Selection Guides are there because they are used in conventional chemical synthesis and in industrial chemicals manufacture to meet contemporary techno-commercial criteria, such as price, assured availability and regulatory approval for use.

None of the Guides, therefore, is designed to guide the search for new solvents. However, more recently, Astra-Zeneca (Diorazio et al. 2016) and Syngenta (Piccione et al. 2019) have reported computer-based interactive solvent selection tools (including additional solvents in the data sets) which complement the Selection Guides.

### Solvent selection and design: empirical database and computational methods

Chemical intuition and experience can be supplemented by developing more systematic methods of solvent selection. Approaches fall essentially into one of two categories (Zhou et al. 2020), either (i) using collections of physicochemical data for sets of molecular compounds to map chemical ‘space’ or (ii) molecular design aimed at molecular species with particular physical or chemical properties. An extensive literature has arisen, including in the search for ‘green’ solvents (Kerton and Marriott 2013a, b, c, d, e, f, g).

Solvency and solvent behaviour are dependent on the physical and chemical characteristics^11^ of both solvent and solute. This relationship has long been studied empirically and fundamentally, both in industry and academia, spanning the work of Snyder, Hildebrand, Hansen, Gutmann, Winstein, Reichardt, Kamlet, Taft, Abrahams, Katritzky and Marcus among many others.^12^

Hildebrand’s solubility parameters, *δ,* (Barton 1991) are based on the idea of the cohesive energy density.^13^ Hansen later allocated to a material, such as a potential solvent, three such parameters parameters, associated with dispersion (*δ*_*d*_), dipolar (*δ*_*p*_) and hydrogen bonding (*δ*_*h*_) inter-molecular interactions (Hansen 1969, 2000). The less one or more of these parameters differ between a solvent and a solute, the more likely it is to dissolve the other or to be miscible or compatible. This can be represented graphically in Hansen ‘space’ (plots of these parameters on separate axes).^14^

Kamlet and Taft (K–T) refined the solvent parameter approach, by dividing the process of dissolution of a solute in a solvent into three notional steps: (i) the creation of a cavity in the solvent to accommodate a molecule of the solute; (ii) the separation of a molecule from bulk (liquid) solute and insertion into the cavity; and (iii) energy release from (attractive) interactions between solvent and solute. These each give rise to a ‘solvatochromic’ parameter^15^ with values determined for a series of solvents and solutes.

Jessop and colleagues have reviewed solvatochromic data for materials of interest in green chemistry as possible replacement solvents (Jessop et al. 2012). These include 83 molecular, predominantly oxohydrocarbon, solvents (67 included in Table S1, the remainder in Table S2, 18) ‘switchable’ (Kerton and Marriott 2013a) solvents and 187 ionic liquids (Kerton and Marriott 2013e). The latter are discussed in “Neoteric solvents: ionic liquids and deep eutectic solvents” section.

One of the earliest reports of a data-led design strategy using sustainability criteria for the identification of substitute solvents dates from 2009, when Estévez (2009) described SOLVSAFE, a European industry-academic collaboration, which applied principal component analysis (PCA; http://www.Wikipedia.org/wiki/Principal_component_analysis). This used a data set of 108 of the most-used solvents and 239 solvent candidates selected from 11 chemical families. Each molecular structure was represented using 52 structural descriptors.^16^ Combining the results from the PCA with predicted toxicological and ecotoxicological profiles allowed potentially low-hazard structures to be identified. The method identified possible replacements for *methyl isobutyl ketone* in chemical synthesis [glycerol formal {a 55:45% mixture (Clark et al. 2017a, b) of 1,3-dioxan-5-ol (RN 4740-78-7) and 1,3-dioxolan-4-methanol (RN 5461-28-8)}]and a solvent for use in paints and varnishes [identifying glycerol isobutyral (2-*iso*-propyl-1,3-dioxolan-4-yl methanol, RN 31192-94-6^17^)].

PCA has also been used (Murray et al. 2016) to project data from ~ 20 physical properties for 136 solvents onto 3 or 4 principal components. From the analysis, *fluorobenzene* and *benzotrifluoride* (C_6_H_4_CF_3_) were identified as possible substitutes for *1,2-dichloroethane*.

Astra-Zeneca (Diorazio et al. 2016) and Syngenta (Piccione et al. 2019) have, more recently, developed interactive tools for solvent selection. Astra-Zeneca (Diorazio et al. 2016) used a database of 272 solvents covering a diverse range of chemical types subject to the practical proviso that they have mp < 30 °C and bp < 350 °C. The scores for seven Safety, Health, Environment (SHE) criteria: Health, Impact in Air, Impact in Water, Life Cycle Analysis, Flammability, Static Potential and VOC Potential, were estimated and normalised to permit a green-amber-red categorisation for each solvent. Data for a range of physical properties and characteristics, either observed or computed, were also included. Instead of placing solvents on a numerical scale associated with a single characteristic such as polarity, both the A-Z and Syngenta methods use PCA to generate simplified ‘maps’ of solvent ‘space’ based on statistical analysis of the data. The Syngenta tool (Piccione et al. 2019) (based on data for 209 solvents, though the full list is not included in or with the paper) is designed to be used iteratively and interactively, using parameters selected according to the judgements of practitioners, to suggest potential solvents for use in laboratory programmes.

A computational method has been used by Durand et al. (2011) to classify 153 representative solvents into 10 categories, similar to those proposed earlier by Chastrette et al. (1985) and others. This modelling tool [Conductor-like Screening Model for Real Solvents, COSMO-RS (Klamt 2005)] has been used to enable comparisons to be made (Moity et al. 2012) between solvents of concern and potential alternatives These were located in a ‘pseudo 3D-space’ using PCA and clustering procedures. The analysis revealed the paucity of, or even the complete lack of, alternatives in some solvent families (e.g. strong electron pair donor bases). Progress using such methods was reviewed in 2014 (Moity et al. 2014a). Artificial intelligence has very recently been used (Sels et al. 2020) to cluster physical properties of 500 solvents in a solvent ‘space’ which can be explored computationally for possible alternatives within the set for a particular solvent. These can then be ranked using SHE criteria.

Results from these computational and multi-criteria analyses may not always match general chemical perception and intuition. This can sometimes lead to novel insights. However, the degree to which such counter-intuitive outcomes are given weight will depend on an assessment of the confidence in the methodology employed, the extent, diversity and completeness of the data sets used and the results of experimental or practical validation.

A wealth of additional studies, too many to survey comprehensively, have focussed more narrowly on the replacement of: (i) a single solvent, such as N*-methylpyrrolidone* (Sherwood et al. 2016) or N,N-*dimethylformamide* (Linke et al. 2020); (ii) a solvent type, such as dipolar aprotics (Duereh et al. 2017) or chlorinated organics (Jordan et al. 2021); (iii) a reaction medium for a particular reaction (Avalos et al. 2006), such as amide coupling (MacMillan et al. 2013), aldehyde reductive amination (McGonagle et al. 2013) or the Delépine reaction (Jordan et al. 2020); (iv) a solvent in a process (Avalos et al. 2006; Fadel and Tarabieh 2019), such as membrane production (Xie et al. 2020); (v) one or more solvents in a series of products (Isoni et al. 2016); (vi) one or more solvents in an application, such as cleaning, (Tickner et al. 2021), lubrication (Dörr et al. 2019), chromatography (Taygerly et al. 2012; MacMillan et al. 2012), or product recovery (López-Porfiri et al. 2020); (vii) one or more solvents in a technology, such as carbon capture (Borhani and Wang 2019), or water-based metal extraction (Binnemans and Jones 2017; Peeters et al. 2020); or (viii) the optimised selection of solvents and anti-solvents in pharmaceutical recrystallization from a defined set of 68 compounds (Tan et al. 2019).

## Solvents and sustainability

The ubiquity, variety and volume of solvents use set those seeking to replace them a complex series of multiple challenges. Despite a wealth of R&D, industrial and academic, progress has been gradual and fragmented. What follows is a brief snap-shot of this progress. The initial focus of green chemistry on the search for supposedly ‘green’ reaction solvents was followed by a gradual shift to a more technology/application-driven approach which, in addition to the intrinsic characteristics of the solvent itself, recognises the importance of how (and indeed where) the solvent is to be sourced, used, managed and disposed of.

The very early occupational health focus on solvent use eliminated (or reduced exposure to) materials of the greatest hazard. Later, particularly in the 1970s and 1980s, limiting solvents use and emissions because of their environmental impact grew to greater prominence. Initially, substitutes were existing articles of commerce, as these were better known and more readily available. The more recent search for new molecular species as candidate solvents has confirmed that most organic materials likely to be technically suitable have long been known^18^ even though little attention may have been given to them as industrial solvents. Despite extensive well-directed efforts, the search identified surprisingly few re-purposed organic liquids as general purpose solvents. Then, after 1998, the green chemistry movement began to explore three under-studied classes of materials, ionic liquids, fluorous fluids and supercritical fluids, widely dubbed ‘green’ or ‘neoteric’ (Seddon 1996) solvents. At the same time, water, the ‘greenest’ of all solvents (Zhou et al. 2019), also underwent a resurgence of academic interest as a reaction medium. A widespread shift from conventional solvents to neoteric has not yet arisen. More recently, the focus has turned to replacing oil, coal and natural gas-sourced solvents and their precursors with those from renewable raw materials, particularly from biomass.

Much of this early work has been covered in depth elsewhere (Avalos et al. 2006; Nelson 2003; Capello et al. 2007; Jessop 2011; Leitner et al. 2010) and brought up to date in recent texts, reviews and articles (Kerton and Marriott 2013a, b, c, d, e, f, g; Welton 2015; Calvo‑Flores et al. 2018; Clarke et al. 2018; Shanab et al. 2013; Shanab et al. 2016; Häckl and Kunz 2018; Clark et al. 2017a, b).

### Green solvents: re-purposed and neoteric

#### Re-purposing known materials

As useful as the Solvent Selection Guides may be, a consideration of the criteria for solvent replacement, listed in Table 2 (Gu and Jérôme 2013), suggests that, in practice, selecting an acceptable substitute solvent is a more complex matter.^19^ Quantitative green chemistry metrics, particularly those developed by John Andraos (2013), highlight the difficulty of fully reconciling technical effectiveness, occupational safety and environmental impact and the impossibility of doing so perfectly.^20^ Judgements about whether one solvent or another is associated with more, or less, waste production, therefore, frequently rely on factors other than the chemical characteristics of the solvent itself (and are generally better evaluated on a case-by-case basis). Indeed, it may well be that such analyses for a process to a particular product or intermediate which uses a so-called green solvent may show, overall, that it is less efficient and more waste-producing than one with a ‘non-green’ solvent.

**Table 2:** Criteria, based on those of Gu and Jérôme (2013), used to judge solvent acceptability

1.	Available on the required scale with a secure long-term source of supply
2.	Technical performance (including solvency) no worse than the equivalent conventional solvent
3.	Stable during use and storage
4.	Low- or non-flammable
5.	Competitively priced
6.	Able to be recycled
7.	Purity appropriate to use
8.	Resource and energy efficient production (preferably life cycle assessed)
9.	Sourced from renewable intermediates and feedstocks
10.	Established acceptable toxicity and ecotoxicity profiles sufficient for regulatory purposes
11.	Fully biodegradable to innocuous products
12.	Meets standards and regulations for transportation

Under the aegis of green chemistry, a mass of research papers proclaim the use of a ‘green’ solvent or the development of a ‘solvent-free’ process (Kerton and Mariott 2013f; Tanaka and Toda 2000). Such research has been usefully summarised by Kerton and Marriott (2013a, b, c, d, g) and others (Welton 2015; Clarke et al. 2018; Shanab et al. 2013, 2016; Häckl and Kunz 2018; Clark et al. 2017a, b). However, too much published material emphasises claimed benefits (by using the following terms in their titles: green(er); clean(er); benign; environmentally friendly; environmentally benign; environmentally green; environmentally acceptable; eco-friendly; and sustainable) while failing to provide the necessary supporting toxicological or ecotoxicological evidence. Often, papers simply ignore the difficulties of scaling up to industrial use. A degree of discrimination, persistence and careful reading is, thus, needed to tease out the undoubtedly valuable work from the less valuable. In addition, too many of these papers ignore completely the use of solvents in product recovery or purification. How significant this is can be seen from an estimate (Kreher et al. 2004) that the volume of solvent used in product work-up and isolation can be 20–100 times that used as the reaction medium. Whether the compounds proposed should (or should not) be considered a ‘green’ solvent can only be judged after an assessment of all the evidence. This must include a consideration of the nature and circumstances of the use to be made of the solvent (whether, for instance, it is to be used in a cleaning formulation sold to the general public or as a reaction medium for a chemical transformation on the large or industrial scale or on the small scale in the laboratory). However, claims continue to appear in the literature that a solvent, or a group of solvents, is inherently ‘green’. One should be sceptical of the concept of a perfect green solvent, capable of the widest applicability in chemical technologies and devoid of environmental impact just as no chemist would today believe in Paracelsus’s ‘alkahest’, the universal solvent of medieval alchemy.

These issues are increasingly recognised by leading practitioners, both in relation to solvent choice and, indeed, to green chemistry itself (Clark 2012), for which ‘12 misunderstandings’, echoing the twelve green chemistry principles, have been identified. Two of these misunderstandings relate specifically to solvents (No. 3: that water is the greenest solvent and No. 6: that involatile solvents are better than volatile ones).

It is also pertinent to note that Fig. 2 suggests that reaction media for use in large-scale chemicals production, while significant, is still only a relatively modest fraction of the total solvent usage, despite having been the dominant focus of green chemistry-driven research (Kerton and Marriott 2013a, b, c, d, e, f, g; Welton 2015; Clarke et al. 2018; Shanab et al. 2013, 2016; Häckl and Kunz 2018; Clark et al. 2017a, b). A valuable additional perspective is provided by Abbott and Shimizu (2019) who focus on coatings and adhesives applications, noting the difference between the physicochemical phenomena involved in the processes of dispersion and solubilisation compared with those of dissolution. They note that these solubilisation phenomena can be described at the molecular level using Kirkwood–Buff (KB) statistical thermodynamics-based theory and are amenable to experimental measurement (Abbott et al. 2017). Related phenomena are relevant to the process of cleaning with solvents (Durkee 2014).

The first major compilation of solvents categorised as green was Nelson’s text of 2003 (Nelson 2003). Nelson’s Table A1 (entitled Green Solvents) lists 659 materials (not all which are included in Table S2). Surprising among these are *benzene*, *carbon tetrachloride*, *carbon disulfide* and *nitromethane*. More recently, two editions have appeared of Wypych and Wypych’s *Databook of Green Solvents* (Wypich and Wypich 2014, 2019) which bring together data sheets for a range of solvents.^21^

Together, Tables S1 and S2 contain 577 molecular materials, with 154 listed in one or other of the Solvent Guides and 423 additional substances used in data sets for computerised solvent selection or design. The great majority of substances cited as ‘green’ (however, defined) are re-purposed long-known materials. Many described as ‘new’ are not. In addition, many described as sustainable are currently available in quantity only from fossil-carbon sources.

Those which have been claimed to be ‘green’ are predominantly oxohydrocarbons (cyclic and acyclic alcohols, esters, carbonates and ethers) with some hydrocarbons and those containing other heteroatoms. These include:

##### **Alcohols**

*ethanol*; *butanol*; *tert-amyl alcohol* (or *2-methylbutan-2-ol*); *glycerol*; and blends of acetone, butanol and ethanol (ABE) (Bankar et al. 2013).

##### **Esters and carbonates**

*methyl acetate*, *ethyl acetate*, dimethyl glutarate (Mouret et al. 2014); *glycerol triacetate*; *ethyl lactate*; *γ-valerolactone*^22^; methyl 5-(dimethylamino-2-methyl-5-oxopentanoate (Lebarbé et al. 2014); *dimethyl carbonate*; and glycerol carbonate (Christy et al. 2018).

##### **Ethers**

*1,3-dioxolane*; *cyclopentyl methyl ether*; iso*sorbide dimethyl ether*; 2,5-dimethylfuran; *2-methyltetrahydrofuran*; *ethyleneglycol monomethyl ether*; *ethyleneglycol dimethyl ether*; triethyleneglycol monoethyl ether [2-(2-ethoxyethoxy)ethanol] (Shakeel et al. 2014); *1,2,3-trimethoxypropane*; and *dihydrolevoglucosenone.*

##### **Others**

*limonene*; farnesane, *benzotrifluoride*; 1,1,1,3,3-pentafluorobutane; piperylene sulfone; *dimethylsulfoxide*; hexamethyldisiloxane; and *N*-(2-methoxy-2-ethoxyethyl)dibutylamine (Samorì et al. 2014).

**Fig. 3:**
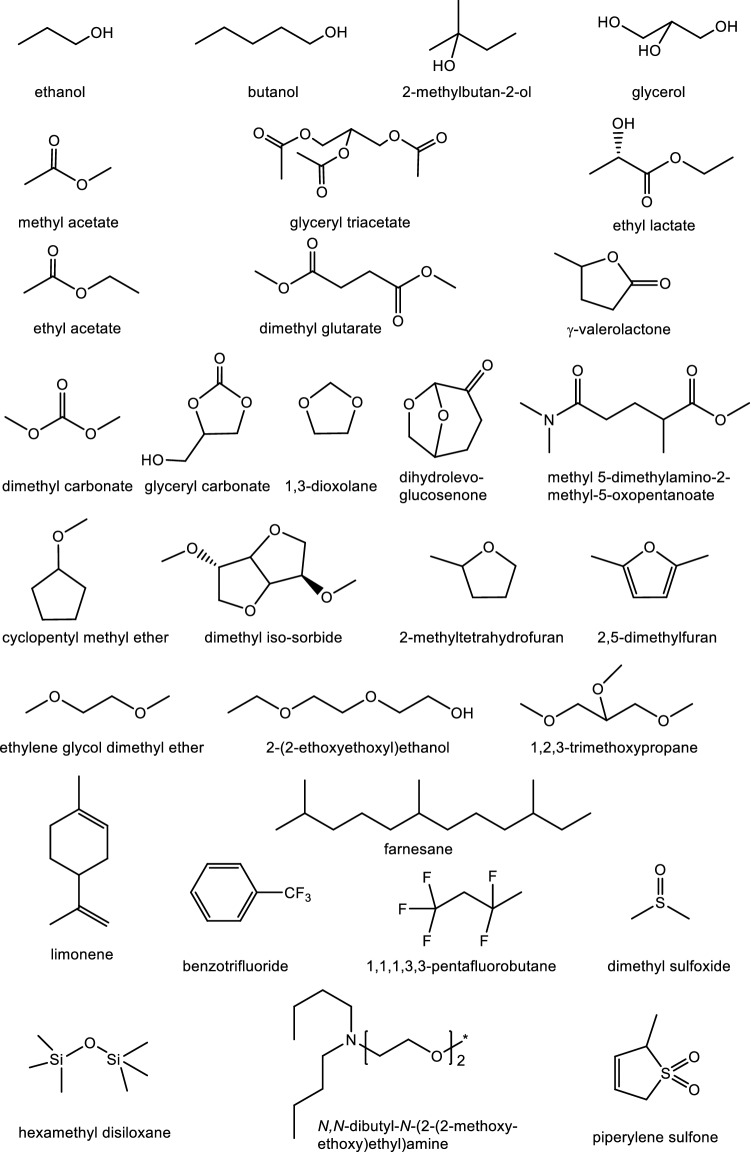
A selection of organic compounds which have been suggested as ‘green’ solvents

A selection is shown in Fig. 3.

A full and proper consideration of the acceptability of using as a solvent any of the compounds listed in a Selection Guide, a tabulation in a text, review or a research paper would represent a significant undertaking even for someone capable of assembling all the evidence, of evaluating its relevance and significance and of making judgements in relation to a specific application. In many cases, substances that have recently become of research interest have very little known about their toxicity (and even less about their environmental impact). Frequently, long-known and widely used materials, such as *dichloromethane*,^23^ are those that have been the best studied and whose toxicology, and the associated risks arising from human exposure, is well understood. A judgement of what might or might not be a suitable solvent is, therefore, no simple matter.

‘Safe’ alternative solvents (however, this may be assessed) that fulfil all critical requirements (as well as being sufficiently technically effective and available on the large scale) are, therefore, difficult to find and to apply. This may explain the results of a survey (Ashcroft et al. 2015) of papers appearing in a leading journal, *Organic Process Research & Development*, during the period 1997–2012.^24^ This concluded that the only replacement solvent to show any significant increase in use was *2-methyltetrahydrofuran*. Indeed, arguments have been put forward for the continuing use, under some circumstances, of certain well-established but less-than-ideal solvents, such as acetonitrile (McConvey et al. 2012).

#### Water

Water exemplifies well the physicochemical basis for the explanation of why a solvent that appears so ‘obviously’ green still struggles to achieve more widespread application as a process solvent (Simon and Li 2012). On the face of it, the use of water has many attractions: it is cheap, readily and widely available, non-toxic and non-flammable. Bearing in mind these obvious advantages (and the fact that it has been available for as long as chemistry and chemical technology have been practised), one may ask why water is not more widely used in chemicals production.^25^ Unfortunately, it is not a good solvent for many organic solutes. This may be addressed by using reaction auxiliaries, such as surface-active agents (Gabriel et al. 2015; Parmentier et al. 2016, 2017), in chemical processing, though this brings its own questions of cost, separability and process operability as well as additional health and environmental impact, direct and indirect. The use of more extreme (therefore, more costly) conditions, such as pressurised hot water (Akiya and Savage 2002; Kawahara et al. 2013) or supercritical water (Bröll et al. 1999), is being researched. However, attempts to develop industrial processes particularly for waste treatment have been hampered by operability and cost issues. On the other hand, the growth in the use of biomass as a feedstock and oxygenated substrates as intermediates in aqueous biocatalytic processing, such as fermentation, is likely to find water increasingly an attractive reaction and process medium.

There are, nevertheless, at least two key problems with water. First, it has a very high heat of vaporisation combined with a very low molecular mass (18 Daltons). So, while a kilogram of toluene requires only 413 kJ to evaporate, water needs more than five times as much (2257 kJ kg^−1^). Such differences may seem unimportant in the context of a research laboratory (where the use of utilities such as electricity, gases of various sorts and cooling water is taken for granted and the associated emissions, gas, liquid or solid, largely ignored). However, in an industrial and commercial context, all utilities usage has to be accounted for and paid for, with energy costs a significant item. Second, to meet regulations concerning the discharge of waste water into rivers and other natural waters the reduction in the concentration of potentially polluting organic solutes often requires very extensive treatment prior to discharge, making the use of water as a reaction medium much less attractive (Blackmond et al. 2007). Indeed, some traditional water-using technologies, such as leather-making, are looking towards organic solvents to reduce water consumption, where water resources are increasingly at a premium (Mehta et al. 2014). On the other hand, in some sectors, such as in the coatings sector, the replacement of organic solvents by water in paint formulations has been a major chemical technology success story^26^ resulting in reduced volatile organic compound (VOC) emissions. However, in large-scale chemicals production there are few examples of water as a reaction medium. The one frequently quoted is the rhodium-catalysed hydroformylation (Kohlpaintner et al. 2001) (Eq. 1), operated^27^ on the 600,000 t y^−1^ scale, taking advantage of the insolubility of the products in the reaction medium to aid their separation.

1 $$ 2{\text{CH}}_{3} {\text{CH}=\text{CH}}_{2} + 2{\text{H}}_{2} + 2{\text{CO}} \to {\text{a}}\;{\text{MeCH}}_{2} {\text{CH}}_{2} {\text{C(O)H}} + {\text{b}}\;{\text{Me}}_{2} {\text{CHC(O)H}} $$

In seeking to extend the scope of such industrial-scale technology, research on water as a reaction medium (Kerton and Marriott 2013b) continues at an elevated level, with a focus on aqueous surfactant mixtures as possible replacements for dipolar aprotic solvents (Gabriel et al. 2015; Parmentier et al. 2016, 2017). Cost-effective substitutes for organic solvents, such as N,N-*dimethylformamide*, N*-methylpyrrolidone*, *sulfolane* and *dimethylsulfoxide,* are important targets, particularly in pharmaceuticals production. Lipshutz, Parmentier and their colleagues (Gabriel et al. 2015; Parmentier et al. 2016, 2017) have sought purpose-designed surfactants as the basis of practical reaction media able to circumvent difficulties such as precipitation, emulsion formation and oiling out that lead to mediocre conversions.

The additional complexity arising from the use of multiple reaction auxiliaries and innovative processing methods, such as ‘tunable’ or ‘switchable’ solvents (Kerton and Marriott 2013a), inhibits rapid and widespread application. The risks associated with simultaneous multiple changes to a technology are considerable. New technology tends to be introduced gradually, first on the more modest (or ‘pilot’) scale, with opportunities to review its benefits and drawbacks before committing large sums of money to implement it on the full scale. It is therefore not too surprising that new applications of the use of water as a reaction medium in pharmaceuticals production are at present^28^ at the stage of using water to make development intermediates on the multi-kg scale (Ikariya and Blacker 2007; Li et al. 2006).

Water can still produce surprises: in 1980, Rideout and Breslow (Rideout and Breslow 1980) observed a significant acceleration of a Diels–Alder reaction in water compared with its rate in octane. In 2005, Sharpless and colleagues (Narayan et al. 2005) saw unexpected rate enhancements when water-insoluble reactants were stirred together in aqueous suspension. Extensive research efforts have followed seeking to understand and exploit these phenomena.

#### Neoteric solvents: ionic liquids and deep eutectic solvents

Widespread large-scale commercial use as yet eludes ‘neoteric’ solvents, such as fluorous fluids,^29^ ionic liquids and eutectic solvents. This is not surprising and does not arise from the ignorance or indifference of the chemical industry. The reasons are relevant and interesting and include the time needed for the implementation of new technology developments and the financial and techno-commercial basis of such developments. Of particular relevance is the degree to which these materials can replace conventional solvents without sacrificing important product or process characteristics, the nature of their toxicity and environmental impact profiles (and the costs of fully establishing them) and market-related factors for the solvents they are aiming to replace, such as cost, assured supply from multiple sources and absence of intellectual property constraints.

Evaporation is an important route for the loss to the atmosphere of a classical molecular solvent (or other liquid) from a chemical process. Its avoidance would make a significant contribution to minimising volatile organic compound (VOC) emissions. Liquid salts or ionic liquids (Freemantle 2010; Kerton and Marriott 2013e),^30^ with melting temperatures near, or even below, room temperature, have negligible vapour pressures. Not only could this avoid solvent loss, it might also make much simpler the separation of volatile products formed in reactions carried out in ionic liquids. Their immiscibility with many conventional non-polar solvents suggests, in addition, that they may aid the extraction of non-volatile products.

As a consequence, these long-known and intrinsically fascinating materials have been the subject of intense recent research and technical interest. Ionic liquids are able to dissolve an impressive range of solutes and have been shown to be capable of mediating a broad range of chemical reactions. Being a combination of anions [A]^n−^ and cations [C]^m+^, the number of permutations [C]_n_[A]_m_ is limited only by chemists’ ingenuity and the melting point below which a salt is considered to be an ionic liquid (by convention 100 °C). From such permutations a very wide range of characteristics can be accessed. A current area of research, that seeks to avoid the life cycle impact of ionic liquids produced from intermediates derived from petrochemical and fossil feedstocks, is the preparation of ionic liquids derivable from renewable resources (Imperato et al. 2007; Tröger-Müller et al. 2017).

The problems standing in the way of the widespread use of ionic liquids as reaction media for large-scale chemical processing, however, are several-fold: (i) their availability and cost. Only a few^31^ are available in quantities of > 1 kg. Few cost less than 20–30 £ kg^−1^ whereas *toluene* or *methyl ethyl ketone*, for instance, cost < 1 £ kg^−1^. A rare example (Chen et al. 2014) of an inexpensive ionic liquid available in bulk is [Et_3_NH][HSO_4_] with a mp of 85 °C and costing < 1 £ kg^−1^; (ii) most ionic liquids are viscous, making mixing and pumping problematic; (iii) their separation, recovery and recycle are technically challenging (Zhou et al. 2018; Mai et al. 2014) because of their very non-volatility, ruling out the conventional method for liquid purification, distillation. In principle, the third of these disadvantages can be circumvented by the use of dissociable ionic liquids (Kreher et al. 2004). [BH]_n_A can be obtained from protic acids, H_n_A, and Brønsted bases, B, or from secondary amines, R_2_NH, and carbon dioxide, giving [R_2_NH_2_][R_2_NCO_2_]. These dissociate on heating, evaporate and then reform on condensation; and (iv) purification of ionic liquids by crystallisation can frequently be problematic, as many (but not all) of them solidify as glasses instead of crystals, and do not undergo the phase change necessary to separate impurities.

Beneficial and successful use of ionic liquids in chemicals manufacture and processing has been summarised by Plechkova and Seddon (2008) and by Gutowski (2017). From time to time, industrial-scale applications are reported (Abai et al. 2015; Tullo 2021) and these continue to be explored (Binnemans and Jones 2017; Sun et al. 2012). There is also, currently, significant interest in the use of ionic liquids in various aspects of biomass processing (Stark 2016). However, taking fascinating and elegant laboratory observations and developing commercially viable processes from them is a major challenge. Widespread use of ionic liquids in large-scale chemical processing appears unlikely. More likely is their use in applications where cost (both intrinsic and that for regulatory toxicity and ecotoxicity testing) is less important than critical aspects of their performance [such as in space (Nancarrow and Mohammed 2017)] or where ionic liquid recovery may be a secondary issue, such as in batteries (Forsyth et al. 2019; Watanabe et al. 2018). Ionic liquids still remain a hot area of research that is capable of regularly producing excitement and surprises. It is possible that some of these may well contribute, indirectly, to more sustainable technologies, not least in energy applications. Nevertheless, a balanced treatment of their strengths and weaknesses in industrial solvents applications should always be insisted upon (Kunz and Häckl 2016).

Ionic liquids are archetypal ‘green’ solvents and continue to be described as such in research papers, reviews and more general perspectives. The benefits arising from the use of a single ionic liquid (or a single class of ionic liquids) have sometimes been extrapolated, without supporting evidence, to cover ionic liquids as a group, though this is seen by leading practitioners to have been an error (McCrary and Rogers 2013; Jessop 2018). Indeed, one recent assessment (Bystrzanowska et al. 2019) concluded ‘*….our results clearly show that the flat assertions on ILs being green solvents are inappropriate and should be avoided*’.

There continue to be relatively few data on which to make a proper judgement concerning the environmental benefits of using ionic liquids (Maciel et al. 2019). Only a limited number of articles have addressed their overall impact or, even more to the point, have compared this with the equivalent impact of conventional molecular solvents. Such methods use life cycle inventory approaches, in which (at the very least) an inventory is made of the materials used and emitted, and the energy consumed, in the production, use and ultimate disposal of an ionic liquid beginning with the primary raw materials from which ionic liquids are made. Cuéllar-Franca et al. (2021) have recently examined the environmental burden of a practical process for post-combustion carbon dioxide capture using life cycle methods. The conventional absorption solvent, 30 wt% monoethanolamine, was found to generate significantly less impact than the ionic liquid 1-butyl-3-methylimidazolium acetate (RN 28409-75-8) despite the better CO_2_-uptake characteristics of the latter. The case for representative ionic liquids, such as 1-butyl-3-methylimidazolium tetrafluoroborate (RN 143314-16-3), being considered ‘green’ when compared with conventional solvents, such as *cyclohexane* (Zhang et al. 2008), is also far from clear cut when such analyses are undertaken.

A related area of research and application involves low-melting liquids [deep eutectic solvents (DES) (Hansen et al. 2021; Smith et al. 2014; Wazeer et al. 2018) and natural deep eutectic solvents (NADES) (Liu et al. 2018)] made from two components, a hydrogen-bond acceptor and a hydrogen-bond donor. These mixtures have three particular advantages: (i) easy production, involving the simple mixing of (usually) two components; (ii) ready availability of a range of low-cost donors and acceptors; and (iii) availability of components whose toxicology, ecotoxicology and biodegradability have been explored.

Abbott, particularly, has developed and applied materials such as the 1:2 (molar) mixture of low-cost salt, choline chloride, [Me_3_NCH_2_CH_2_OH]Cl, with *ethylene glycol* (Abbott et al. 2006) in industrial electro-polishing of stainless steel, instead of the conventional mixed sulfuric/phosphoric acids. The electrochemical recovery of high purity copper from chalcopyrite copper sulfide mineral uses a mixture of two DES [choline chloride-oxalic acid (20%) and choline chloride-ethylene glycol (80%)] without forming either sulfur dioxide or hydrogen sulfide (Anggara et al. 2019). The solvo-metallurgical recovery of lithium and cobalt from spent end-of-life lithium ion battery cathodes using 2:1 (molar) choline chloride-citric acid DES has also been investigated (Peeters et al. 2020).

### Biosolvents or bio-derived solvents (Gu and Jérôme 2013; Soh and Eckelman 2016; Clark et al. 2015; Calvo‑Flores et al. 2018; Grundtvig et al. 2018; Dapsens et al. 2012)

Biomass-accessible chemicals have long been known and some have been used as solvents. These include natural products obtained from wood, seeds and cereal crops (or from wastes associated with their production^32^) and their derivatives. Many of these products tend to be mixtures of variable composition depending on the source. This can be problematic when considering their use in chemical synthesis and manufacture, particularly in the highly regulated pharmaceuticals industry. This difficulty might be avoided if a limited number of bio-derived commodity chemical ‘platforms’ (Werpy and Petersen 2004) could be identified to provide routes to products of uniform and specified purity. However, trying to replace a commodity product, such as *hexane* (Moity et al. 2014a) or *toluene* (Paggiola et al. 2016) with a bio-derived equivalent, such as *limonene* or pinene can run into other problems.

Nevertheless, availability of chemicals from renewable feedstocks is becoming of increasing interest, either directly by conversion of a biomass-derived component such as lignin pyrolysis oil (Mudraboyina et al. 2016) or as the source of versatile precursors to downstream products [‘platform’ chemicals (Werpy and Petersen 2004)]. Platform chemicals may provide potential routes both to existing solvents and to new ones. In addition, their potential accessibility via water-based biotechnological processes, such as fermentation, operated at temperatures much lower than equivalent chemo-catalytic routes, may bring environmental benefits that arise as much from how (and from what) the product is made as from how it is used. Making platform chemicals or individual products directly from biomass itself or from one of its major components, such as lignocellulose or carbohydrates, is now a major research topic (Winterton 2021a).

The best-established high-volume process for production of a commodity chemical (primarily used as fuel additive) is sugar fermentation to ethanol. The possible use of ethanol as a feedstock to produce downstream chemical products is an active research area. Two additional examples, using both chemical and biotechnological processes, illustrate the use made of carbohydrate feedstocks. The fermentation of sugar cane using a genetically engineered yeast produces (instead of ethanol) a C15 unsaturated hydrocarbon, β-farnesene (RN 18794-84-8), Me_2_C = CHCH_2_CH_2_C(Me) = CHCH_2_CH = C(Me)CH = CH_2_. This can be chemo-catalytically hydrogenated to farnesane, C_15_H_32_ (RN 3891-98-3), a diesel substitute. Amyris, the company which built a plant to produce farnesane in Brazil (close to the source of sugar-cane), was awarded a 2014 Presidential Green Chemistry Award (http://www2.epa.gov/green-chemistry/2014-small-business-award) for its development. However, subsequent reports highlight the considerable challenges, technical and economic, associated with the manufacture of biomass-derived products on the large industrial scale to the extent that Amyris was reported (Bomgardner 2017) to be selling the plant to DSM.

The second example exemplifies a major technological target (Jessop 2011), viz*.* the search for low-impact, low-toxicity, cost-effective and readily available alternatives to polar aprotic solvents from bio-derivable precursors or raw materials. Levulinic acid is a platform chemical, which can be co-produced from acid treatment of sugars and starch and, more recently, has been synthesised from lignocellulose. Levulinic acid can provide routes to a series of downstream products (at least on the research or small technical scale) including *γ-valerolactone*, *2-methyltetrahydrofuran* and alkyl levulinates. A 3-step synthetic route from bio-derivable alkyl levulinates leads to a series of *N*-substituted 5-methylpyrrolidones (Barbaro et al. 2020). The *N*-heptyl derivative (RN 69343-70-0) has a bp of 94–95 °C at 1.0 torr. Lignocellulose can also give furfurylacetone (a solid with mp 86–87 °C) whose hydrogenation and deoxygenation lead to 2-butyltetrahydrofuran (RN 1004-29-1) (Strohmann et al. 2019), proposed as a biofuel, though with physical characteristics, including a bp of 159–160 °C, which might also lead to its consideration as a solvent.

**Scheme 1:**
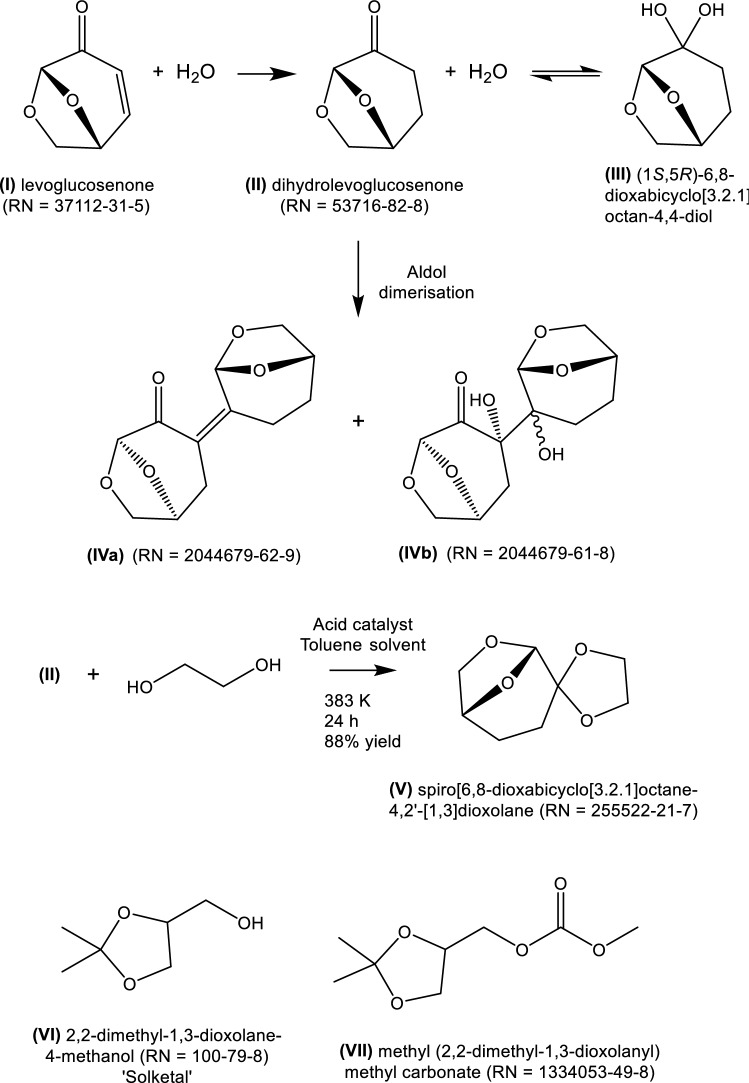
Solvents derivable from biomass via levoglucosenone or glycerol

In 2014, Clark and colleagues proposed in a preliminary communication (Sherwood et al. 2014) that *dihydrolevoglucosenone* (**II**) (Scheme 1) had solvency and other characteristics which made it a candidate substitute for dipolar aprotic solvents, such as N,N-*dimethylformamide*, N-*methylpyrrolidone* and *sulfolane*. The chemical and physical properties and technical applications of **II** have now been studied extensively (Camp 2018), such that its strengths and limitations as a solvent are better known. In addition, evidence has become available of its toxicological and ecotoxicological promise leading to initial regulatory approval (Camp 2018; De bruyn et al. 2019).

*Dihydrolevoglucosenone* can be obtained from chemo-catalytic hydrogenation (Krishna et al. 2017) of levoglucosenone (**I**), itself obtained in low yield from the pyrolysis of cellulose. This is the basis of the Furacell™ manufacturing process for **II**, sold under the trade name, Cyrene®, carried out in a 50 t y^−1^ pilot plant operated by the Circa Group in Tasmania (Richardson and Raverty 2016). A recent report (McCoy 2020) suggests that a consortium funded by the European Union is proposing to build a 1000 t y^−1^ plant in France.

*Dihydrolevoglucosenone* readily equilibrates with water to form the geminal diol, [(1*S*,5*R*)-6,8-dioxabicyclo[3.2.1]octan-4,4-diol (**III**)] (De bruyn et al. 2019). It can also be sensitive to inorganic bases which under some conditions leads to the formation of a solid dimer (**IV**) (RN 2044679-62-9) (Wilson et al. 2016), rendering solvent recovery and reuse problematic (Wilson et al. 2019). These characteristics may limit its applicability, particularly in biocatalytic applications.

The research phase to new biosolvents has many challenges, illustrated for lignocellulose- and glycerol-derived products. *Dihydrolevoglucosenone* was one of 164 compounds derivable from levoglucosenone. Clark and colleagues (Pacheco et al. 2016) subjected this set to a coarse selection method, based on melting point, and Hansen and Kamlet–Taft (K-T) solubility parameters. Fifteen of the 164 were considered as possible replacements for *dichloromethane* and N-*methylpyrrolidone*. From a series of ketals derived from *dihydrolevoglucosenone*, spiro[6,8-dioxabicyclo[3.2.1]octane-4,2′-[1,3]dioxolane] [(**V**); RN 155522-21-7] was identified as having (K-T) parameters which were a reasonable match for those of *dichloromethane* and N-*methylpyrrolidone*. **V** was found to perform comparably to N,N-*dimethylformamide* and N-*methylpyrrolidone* in a representative nucleophilic aromatic substitution and a palladium-catalysed carbon–carbon coupling reaction. Unfortunately, **V** is solid at room temperature, with a mp of 60 °C (Pacheco et al. 2016). A similar methodology (Jin et al. 2017) was employed using glycerol as the bio-derivable precursor. Solketal [(**VI**); *2,2-dimethyl-1,3-dioxolane-4-methanol*; RN 100-79-8] can be obtained from glycerol and acetone (Nanda et al. 2016). The report from Jin et al. identified methyl(2,2-dimethyl-1,3-dioxolan-4-yl)methyl carbonate [(**VII**); RN 1354053-49-8)] formed from *solketal*. **VII** is high-boiling (232 °C), melting at -7 °C, and was found to be mutagenic in an Ames assay.

Other work has explored two further aspects related to the use of biomass or biomass-derived precursors. The first concerns the selection of solvents (using COSMO-RS) for processing biomass and the conversion of biomass-derived materials, such as biphasic dehydration of sugars to 5-hydroxymethylfurfural and *furfuraldehyde* (Esteban et al. 2020). The second considers biomass and derived compounds as possible sources of new or existing products. For example, one study (Moity et al. 2014b) has sought new solvents from a bio-based precursor (itaconic acid [CH_2_=C(CO_2_H)CH_2_CO_2_H]; RN 97-65-4) using computer-assisted organic synthesis. The methodology employed 53 basic transformations to generate an initial series of 40 candidate derivatives using simple inorganic or organic co-reactants, such as methanol, acetic acid, acetone or glycerol, methylamine. 14 of the 40 are known compounds, some also available from starting materials other than itaconic acid. However, only one of these, 2-methylenebutan-1,4-diol is a liquid (RN 55881-94-2; mp 8 °C).

Bio-derived or potentially bio-derived^33^ products have been investigated in the search for wholly new materials to replace solvents of concern. Biomass-derived, or potentially biomass-derived, solvents have also been used as media in which to carry out CO_2_-based carboxylations (Gevorgyan et al. 2020). These included polar aprotic biomass-derived ethers [*2-methyltetrahydrofuran*, acetaldehyde diethyl acetal, *isosorbide dimethyl ether*, *2,2-dimethyl-1,3-dioxolane-4-methanol* (Cyrene®), eucalyptol (also known as 1,8-cineol), Rose oxide] and esters *(γ-valerolactone, diethyl succinate*, *ethyl acetate*), as well as non-polar aprotic unsaturated terpenes and their derivatives [γ-terpinene, α-pinene, *(R)-(* +*)limonene*, *p-cymene*]. Acetaldehyde diethyl acetal, *isosorbide dimethyl ether,* eucalyptol, Rose oxide (tetrahydro-4-methyl-2-(2-methyl-1-propen-1-yl)-2*H*-pyran; RN 16409-43-1)*, diethyl succinate,* γ-terpinene and α-pinene are prominently highlighted as  solvents. However, these are not previously unknown compounds. Indeed, two are found in Table S1 indicating their industrial use as solvents and four in Table S2.

An exploratory comparison has been published (Pellis et al. 2019) of the conventional solvents, *toluene* and *tetrahydrofuran*, with four bio-derived (or potentially derived) solvents, 2,2,5,5-tetramethyltetrahydrofuran, *2-methyltetrahydrofuran*, 2,5-dimethyltetrahydrofuran and 3,3-dimethyl-2-butanone (pinacolone). The six solvents, conventional and bio-based, all performed similarly in a model enzyme-catalysed polyesterification between dimethyl adipate and 1,4-butanediol with, overall, pinacolone the best performer.

Biomass is a primary feedstock of greater complexity and intractability (Winterton 2021a) compared with those from petrochemical sources, such as oil or natural gas. Reversing the replacement of historically important bio-based chemical production technologies by petrochemicals with modern bio-sourced equivalents represents huge challenges. These start with the production, handling, transport and processing of biomass on the large, particularly the global, scale. These add to costs, are energy consuming and will slow the pace of implementation of a new industrial chemical supply infrastructure.^34^ Two additional aspects of converting biomass to industrial chemicals, particularly in relation to the reduction and possible elimination of emissions of carbon dioxide, are evident: (i) the amount of ammonia-based fertiliser needed for biomass growth (currently dependent on fossil carbon both for ammonia production and for fertiliser application and biomass harvesting) and (ii) the poor overall material and energy efficiencies for converting biomass, via multi-step process chains, to the desired products (Clark et al. 2015; Winterton 2021a). These questions go to the heart of the renewability of biosolvents. The degree of renewability will be based upon, among other things, an assessment of the extent of fossil-carbon materials and energy used, directly or indirectly, in the biosolvent production cycle.

### Solvents from other renewable sources

There are few possible large-scale sources of organic solvents, or their precursors, other than petrochemical and biomass carbon. However, both fossil carbon and renewable carbon arise by photosynthesis ultimately from carbon dioxide, water and sunlight. Can the latter be used directly to make chemicals, including solvents, on the industrial scale?

Carbon dioxide is a component of the atmosphere (currently 412 ppm, steadily increasing from its pre-industrial concentration of ca 280 ppm). It is a product of the combustion of fuel and is emitted in industrial waste streams. Its capture and sequestration for reuse are a major test of the so-called circular economy (Winterton (2021a). It is possible, in principle, to remove carbon dioxide from the atmosphere by so-called Direct Air Capture or from the more concentrated industrial process streams. In either case, the technological and economic challenges are immense. Carbon dioxide currently has applications in the food and drinks industries and in enhanced oil recovery. It is readily compressed (> 31.0 °C, > 72.8 atm) to a supercritical fluid (Kerton and Marriott 2013c) which finds use in supercritical extraction, such as the removal of caffeine from coffee and in dry cleaning. Supercritical CO_2_ is also under active R&D investigation in the production of fine chemicals and pharmaceuticals.

The use of carbon dioxide as a chemical feedstock is limited, largely confined to making urea and methanol. Interest in solvents derivable from CO_2_ arises particularly from the production of carbonate esters, such as *dimethyl carbonate*. However, large-tonnage production currently uses phosgene as the raw material of choice (Andraos 2012). Development of routes avoiding the latter is a particular focus. Indeed, a process making several thousand tons per year of dimethyl carbonate by oxidative carbonylation of (currently fossil-carbon-derived) methanol has been operated by EniChem (Keller et al. 2010) for nearly 30 years (http://www.ihsmarkit.com/products/chemical-technology-pep-reviews-dimethyl-carbonate-from-methanol.html). However, direct routes to carbonate esters sourced wholly from carbon dioxide itself remain at the research and experimental stage. Unfortunately, all such reactions of CO_2_ leading to compounds with C–H bonds (the bulk of organic solvents) are severely energetically disfavoured. To operate industrially, most require the large-scale availability of elemental hydrogen (or an equivalent source of reducing power) from non-fossil-carbon sources. Nevertheless, a major current initiative focusses on methanol from renewable sources as a central component of the so-called methanol economy (Olah et al. 2018), encompassing transportation fuel and chemicals production, as well as providing a readily transportable form of stored energy. This would entail a major change in energy supply infrastructure requiring immense investment. Whether, in the transition, free-standing operations on a smaller scale might provide routes to downstream products, including solvents, they would still need to raise finance and build the necessary infrastructure once successful production technology had been demonstrated. Commodity organic solvents directly from carbon dioxide are not a short or medium term prospect.

## Conclusions

Solvency and solvation are important chemical phenomena. Solvents come from all chemical classes and have a wide and disparate range of industrial and domestic applications. The consequences of associated solvent losses motivate efforts to find less-impacting substitutes.

Bearing in mind the multiplicity of functions required of solvents, their selection in any particular set of circumstances is a complex process. Choice of solvent is always a compromise governed by the specific circumstances of its particular use. Key factors to optimise and reconcile include relevant physicochemical, technical, cost, availability at scale, regulatory, safety and sustainability criteria.

Since the late 1990s, solvents have become a major focus of green chemistry. In addition to studies of ‘solvent-free’ processes (Kerton and Mariott 2013f; Tanaka and Toda 2000), green chemistry gave rise to the idea of the green solvent, primarily as a medium in which to carry out chemical reactions or processing. An extensive literature describing the discovery and use of so-called green solvents has arisen. An evaluation of this body of research allows the following to be concluded:

Listing the basic characteristics of an ideal green solvent has set targets to aim for, though, may have also given rise to the belief that it is possible to design a green solvent without proper regard to its particular use, especially on the industrial scale. Because the perfect meeting of all these requirements for universal use (an ‘alkahest’) is unattainable, no solvent can be considered inherently or universally ‘green’. Despite this (and despite the efforts of leading practitioners), individual solvents and classes of solvents continue to be described as green, even when they do not (and may not in the future) meet important practical-use criteria.

Both empirical and molecular design methods are available to assist solvent selection. These have included various ways of balancing cost effectiveness, occupational safety and environmental impact. However, two difficulties arise: first, in assigning the weighting of the various evaluation criteria and second, in the extremely patchy availability of relevant and reliable data.

Even after many studies using a range of approaches, few previously unreported conventional organic compounds have been applied on the industrial scale as reaction media. In fact, the majority of such substances are re-purposed long-known materials (including water). Many described as ‘new’ are not. In addition, many described as sustainable (particularly oxygenated compounds such as esters and ethers) are currently available in quantity only from fossil-carbon sources.

A focus of green chemistry has been on so-called ‘neoteric’ solvents, such as ionic liquids, deep eutectics, fluorous fluids, supercritical fluids and water. Creative research has produced novel insights. However, widespread and large-scale technological application has so far been modest, with few replacement reaction media being used on the industrial scale. While this is so, an increasing number of relatively small-scale applications in new high technology areas (such as in electronics fabrication and battery electrode recycling) have arisen.

Biomass or biomass-derived commodities are under intense investigation as feedstocks for chemicals, fuel and energy hitherto provided from petrochemical sources. Water (with its own availability problems) is increasingly likely to be the solvent of choice in biomass processing, particularly those involving carbohydrate components. Studies include efforts to source ‘biosolvents’ from biomass. In principle, most existing solvents in large-scale use, both oxohydrocarbons and hydrocarbons, are accessible this way. However, in practice, there are currently relatively few such bio-sourced products available in volume. Much of this arises from complexities in the supply, handling and processing of biomass.

Sourcing chemicals, including solvents, from carbon dioxide recovered from industrial waste streams or from the atmosphere is an active area of multidisciplinary research, though one representing only a long-term prospect.

It is probably better, now, to talk in terms of a sustainable solvent rather than one that is new or green, that is one that has undergone an assessment, preferably using life cycle assessment methodology, of the impact (toxicological, ecotoxicological, environmental, resource depletion, economic) of its production chain (all the way from its primary feedstock), its formulation and use and its fate or ultimate disposal.

## Supplementary Information

Below is the link to the electronic supplementary material.

Supplementary file1 (DOCX 63 kb)

Supplementary file2 (DOCX 108 kb)

## Data Availability

Data sharing is not applicable as no datasets were generated or analysed for this article.

## Footnotes

1 Many hundreds of chemical compounds (not to mention their mixtures) have been reported as possible solvents. These have been brought together in Tables S1 and S2. The tabulated content is explained in more detail when the tables are first mentioned in the text. However, because of their length, they are included in electronic form to be found in Supplementary Information as Online Resources 1 and 2, respectively, each with some additional material to aid the reader to navigate and use them.

2 Such choices are best illustrated with an example. A very valuable painting by Mark Rothko, ‘*Black on Maroon*’, was subjected to an act of vandalism that left it marked with graffiti ink What should be the key criterion used in selecting a cleaning fluid to remove the ink in this case? Should cost be a factor, considering that the painting last changed hands for $US 27 M? Should only ‘green’ solvents be used? Or should effectiveness be the prime criterion (that is, an ability to remove the ink completely without damaging the underlying art work in any way). After the solubility characteristics of the ink (the solute) were determined (“Solvent selection and design: empirical database and computational methods” section), combinations of various non-aqueous solvents were proposed for initial investigation. A mixture of *benzyl alcohol*, (see footnote 3) C_6_H_5_CH_2_OH, and *ethyl lactate*, CH_3_CH(OH)C(O)OC_2_H_5_, was finally chosen for use in the restoration (which cost an overall £200 K) (Barker and Ormsby 2015). See Prati et al. (2019) and Baij et al. (2020) for other recent publications concerning the role of solvents in the restoration of works of art.

3 A named molecular species is *italicised* if it is listed in Table S1 (Online Resource 1), that is, included in one or more Solvent Selection Guides discussed in “Solvent guides from the pharmaceutical industry” section. One that is found in Table S2 (Online Resource 2), that is taken from one of the databases discussed in “Solvent selection and design: empirical database and computational methods” section, or from one of a number of papers highlighting a particular compound as a green solvent, is underlined.

4 It is worth noting at this point that inclusion in a Solvent Selection Guide discussed in “Solvent guides from the pharmaceutical industry” section should not be taken as indicating a compound is necessarily ‘green’, sustainable or safe to use. In particular, note should be taken of newer hazard information coming available after the Guides were assembled and published.

5 Two very recent studies of the design and selection of solvents used in the isolation of paracetamol (Ottoboni et al. 2021) and ibuprofen (Watson et al. 2021) highlight the associated complexities.

6 An excellent industry perspective of solvent selection can be found in the article by Gani et al. (2006).

7 See classical practical texts such as those of Vogel et al. (1989) and Riddick et al. (1986).

8 The use of tin instead of lead in perovskite solar cells (PSCs) could reduce toxicological and environmental hazards associated with production and use. However, *DMSO*-containing solvents used to process tin-based PSCs lead to loss of performance associated with oxidation of tin(II) to tin(IV). Di Girolamo et al. (2021) have recently developed *DMSO*-free solvent systems for processing formamidinium tin triiodide perovskite based on 16 non-*DMSO* compounds (and 66 mixtures) selected from an initial set of 80 candidates. Characterisation and performance tests identified 6:1 *N,N*-diethylformamide/*N.N′*-dimethylpropyleneurea as a promising combination.

9 Please also see Table S2 (Online Resource 2). This lists a further 417, mostly molecular, solvents + 6 ionic liquids. Most of these have been assembled from various compilations and data sets used in the computerised identification and selection of solvents with particular characteristics and are discussed in “Solvent selection and design: empirical database and computational methods” section. Some materials cited as green in additional papers [and from one website (http://www.xftechnologies.com/solvents)] are also included (Bankar et al. 2013; Barbaro et al. 2020; Bauer and Kruse 2019; Bergez-Lacoste et al. 2014; Byrne et al. 2017; Byrne et al. 2018; Calvo‑Flores et al. 2018; Christy et al. 2018; Clark et al. 2017a; Cseri and Szekely 2019; Di Girolamo et al. 2021; Diorazio et al. 2016; Driver and Hunter 2020; Estévez 2009; Gevorgyan et al. 2020; Ho et al. 2020; Hu et al. 2014; Jessop et al. 2012; Jin et al. 2017; Kobayashi et al. 2019; Lee et al. 2017; Linke et al. 2020; Marcel et al. 2019; Moity et al. 2014a, b; Mudraboyina et al. 2016; Murray et al. 2016; Pellis et al. 2019; Piccione et al. 2019; Qian et al. 2021; Samorì et al. 2014; Shahbazi et al. 2020; Shakeel et al. 2014; Sherwood et al. 2016; Strohmann et al. 2019; Tobiszewski et al. 2015; Vinci et al. 2007; Wypych and Wypych 2014, 2019; Yang and Sen 2010; Yara-Varón et al. 2016; Zhenova et al. 2019).

10 The Chemical Abstracts Service Registry Number (CAS RN) is a unique identifier for chemical substances the characteristics of which have been published and abstracted by the Chemical Abstracts Service of the American Chemical Society. The use of this number as a search term in the SciFinder® data base (http://www.scifinder-n.cas.org) provides links to associated chemical literature, key physical and chemical properties and other useful information. The unique identifier is particularly useful when an individual compound has a number of common, technical and scientific names. Where known, RNs are listed in all the compound tabulations in the text.

11 An extensive analysis (Katritzky et al. 2005) of 127 solvent scales and 774 solvents concluded that all of the different ways in which molecular structure affects intermolecular interactions in liquids can be associated with cavity formation, electrostatic polarisation, dispersion and specific hydrogen-bond formation.

12 For a list of 183 different solvent scales, see Table 1 in a review on solvent polarity by Katritzky et al. 2004.

13 The cohesive energy density (CED) is the amount of energy needed to remove (to an infinite distance) a mole of solvent molecules from their neighbours. This equates to the heat of vaporisation of the solvent (*H*_*v*_ – *RT*) (where *R* is the gas constant, and *T* is the absolute temperature) divided by the molar volume, *V*_*m*_. The Hildebrand solubility parameter,*,* equals (CED)^0.5^. Hildebrand proposed that dissolution, miscibility or swelling would be more likely between materials with similar δ.

14 A related approach developed by Snyder [and reviewed by Barwick (1997)] categorises solvents into 8 different classes.

15 Solvatochromic parameters, so-called because they are obtained from spectroscopic studies using probe solutes, are one of a series of related solvent parameters, summarised by Marcus (1993). A solubility characteristic, XYZ, can be represented by linear combinations of energy contributions associated with K–T’s three energy terms: XYZ = XYZ_0_ + cavity term + dipolar term + hydrogen bonding terms. K–T introduced a series of parameters (solvatochromic parameters) for solvent (subscript 1) and solute (subscript 2). These include a dispersion term (*δ*_*d*_^2^)_1_, dipolar terms (*)_1_ and (*)_2_ and hydrogen bonding donor and acceptor terms, depending on whether the interaction is between a donor solvent and acceptor solute (α)_1_ and (β_m_)_2_ or vice versa, (β)_1_ and (α_m_)_2_. The effects of different solvents on the properties of a single solute can be represented by equations such as (XYZ = XYZ_0_ + *h*(*δ*_*d*_^2^)_1_ + *s*(π*)_1_ + *a*(α)_1_ + *b*(β)_1_), where *h*, *s*, *a* and *b* are coefficients independent of the solvent. Similar relationships can be used to represent the behaviour of different solutes in a single solvent. For a useful short explanation of the K–T approach, including a tabulation of parameter values, see Kamlet et al. 1986.

16 Principal component analysis is a method which transforms data from a large number of variables (such as structural descriptors) into a smaller set, while retaining most of the information of the larger set. As a result, the data are easier to visualise and explore. In this case, a 52-dimensional chemical space was ‘projected’ into a two-dimensional space.

17 This compound was first mentioned in Chemical Abstracts over 80 years ago (Trister and Hibbert 1937) and last cited in 1983. More recently, it was patented for cleaning, polish-remover and coatings applications and as a fuel additive. It is structurally similar to *2,2-dimethyl-1,3-dioxolan-4-yl methanol* (RN 100-79-8), obtained from acetone and glycerol, and sold for similar applications as *Solketal*.

18 While this was true of solvents, the search for non-ozone-depleting refrigerants, blowing agents and aerosol propellants, following international agreement of the Montreal Protocol in 1987, saw the accelerated testing, production and use of new fluorohydrocarbon products, essentially from scratch, in a global collaboration by major chemical companies.

19 Tickner et al. (2021) report that the development by Eastman Chemicals Co. of butyl 3-hydroxybutyrate (RN 53605-94-0) as the industrial cleaner, Omnia™, began with a database of *ca.* 3000 possible candidates.

20 The lack of integration between green chemistry and the wider effort searching for alternative chemicals has also been discussed in a recently published perspective (Tickner et al. 2021). The perspective analyses factors are behind this lack of integration. Among these is a need by researchers seeking alternatives to appreciate end-use application and associated critical technical, toxicological and sustainability characteristics.

21 The Wypychs’ compilation of data sheets brings together a wide range of useful technical, regulatory and chemical data. 312 products are listed in at least one of the editions. The products are classified according to the solvent types commonly identified as being green. However, examination of the allocation and the nature of the materials included reveals how vague and arbitrary are the definition of a green solvent. 175 are branded products which are mainly mixtures or formulated blends, some of precise, others of variable or undefined, composition, usually sold to meet a technical specification. Unique components having a Chemical Abstracts Service Registry Number (see footnote 10) are included in Table S2 (Online Resource 2). Some materials, likely to be generally impractical as solvents, such as tetrafluoromethane, a gas with a very low boiling point, or those which are solids at room temperature, are excluded. Mixtures where little or no compositional information is provided with the CAS RNs, as is the case with many fatty acid methyl esters, are not included either. Many of the mixtures are biomass-derived and are, as a consequence, likely also to be subject to source-related compositional variation. However, formulated blends having a RN containing identified pure components are included.

22 The availability and use as a solvent of closely related *γ-butyrolactone* (96-48-0), discussed earlier in relation to solar-cell fabrication (Jeon et al. 2014), pose a dilemma because it is readily metabolised to the recreational drug GHB.

23 Two surveys of research papers which have appeared in a leading journal, carried out slightly differently, but both relevant to changes in solvent usage in pharmaceuticals and medicinal chemistry research over 1997–2012 (Ashcroft et al. 2015) and 2009–2019 (Jordan et al. 2021), respectively, both show the continuing importance of *dichloromethane*. The latter survey placed *dichloromethane* as the most widely used research solvent as recently as 2019. In 2019, *2-methyltetrahydrofuran* was placed 12^th^ of 25 and *dimethyl carbonate* 23^rd^ of 25. Neither was in the top 25 in 2009.

24 An unpublished update reviewing papers between 2012 and 2016 (A. S. Wells, personal communication) suggests that little had changed, with growth in *2-methyltetrahydrofuran* use continuing and a small number of examples of *cyclopentyl methyl ether* use being noted. Very few uses of neoteric solvents were evident.

25 Even if water is little used as a reaction solvent, it is still used in large volumes industrially for extraction, washing and as a coolant. It is rare, however, for water use to be taken into account in estimating material use efficiencies. It is excluded from Sheldon’s E-factor and widely used industrial metrics, such as Process Mass Intensity. Other means are needed to account for such water use (Lipshutz et al. 2013).

26 …and one whose implementation in globally marketed products was well advanced before the advent of green chemistry.

27 Since 1984—also pre-green chemistry.

28 It is difficulty to be precise or certain as industry, to avoid giving away its intentions to its competitors, is usually guarded about what it makes public concerning its developmental activities.

29 Processing using supercritical fluids is a well-established technology seeing a recent resurgence of research interest under the green chemistry banner (Kerton and Marriott 2013c). Similarly, industrial use of fluorinated fluids has a history of industrial use as working fluids in refrigeration, solvents, blowing agents, aerosol propellants and fire-fighting chemicals. A range of new fluorinated products [including ‘fluorous fluids’ (Kerton and Marriott 2013d)] has been synthesised for similar specialist applications. Eight fluorine-containing materials are listed in Table S1 (Online Resource 1). 19 other fluorine containing molecular materials are listed in Table S2 (Online Resource 2), six of which arose during the green chemistry era. These have been patented for specialist cleaning applications. However, they have yet to find widespread large-volume use in chemical processing. There have also been concerns about the environmental impact of related products.

30 What is, or is not, an ionic liquid is somewhat arbitrary. See Abbott et al. (2015) and, for my own view, see Winterton (2015).

31 Table S2 (Online Resource 2) lists only six such salts, a choline derivative, 2 phosphonium and 3 imidazolium compounds: choline acetate, tributyltetradecylphosphonium chloride, (tetradecyl)trihexylphosphonium chloride, 1-ethyl-3-methylimidazolium bis(trifluoromethylsulfonyl)imide and bis(fluorosulfonyl)imide, and 1-butyl-3-methylimidazolium tetrafluoroborate. However, most early compilations pre-date the promotion of ionic liquids as green. Since then, a very large number of ionic liquids have been reported, their properties and applications investigated. My own survey (Winterton 2015) lists ~ 750 salts for which a single crystal structure has been deposited with the Cambridge Structural Database of the Cambridge Crystallographic Data Centre.

32 *Furfuraldehyde* (furfural) has long been known (Brownlee and Miner 1948) as a product readily derivable from waste oat hulls, a by-product in the production of food and animal feed. *Furfuraldehyde* has recently been reported as a precursor to two possible replacements for *acetone* and *methyl ethyl ketone* in the solubilisation of epoxy resin prepolymers (Bergez-Lacoste et al. 2014), namely pentyl 2-furoate (RN 4996-48-9) and 1-(2-furanyl)-4-methyl-1-penten-3-one (RN 4196-97-8). The methyl, ethyl and isopropyl esters of 2-methyl 5-furancarboxylic acid (RNs 2527-96-0, 14003-12-4, 7042-05-9, respectively) are currently being marketed (http://xftechnologies.com/solvents) as alternatives to *methyl ethyl ketone*, *tetrahydrofuran* and N*-methyl-2-pyrrolidone*.

33 This distinction is particularly important when assessing claims that commercial products contain bio-based components or are derived from biomass-sourced precursors. This arises especially when there are petroleum-derived materials which in all other respects are indistinguishable from the bio-derived material. In addition, many research papers investigating the chemistry of substances described as bio-derived are often vague as to the precise provenance of the materials used. For these reasons, one regulatory agency, the European Committee for Standardisation, has issued technical standards for testing of bio-based solvents and for determining the bio-based content of bio-based products [European Committee for Standardisation (CEN) 2015, 2017)].

34 The use of existing commercially available solvents from fossil-carbon sources in new applications, such as replacements for dipolar aprotics in fine chemicals manufacture, avoids some of the questions of availability and supply. Commercially available Rhodiasolv®Polarclean (Randová et al. 2016) is marketed by Solvay to solubilise agrochemicals. Technical grade Polarclean has been investigated in both polysulfone (Dong et al. 2020) and PVC (Xie et al. 2020) membrane fabrication as an alternative to the N,N*-dimethylacetamide*, *dimethylformamide* and N*-methylpyrrolidone* currently employed. Polarclean is made from 2-methylglutaronitrile (from butadiene hydrocyanation) and, dependent on the source of the sample, contains variable (85–95%) amounts of methyl 5-(dimethylamino)-2-methyl-5-oxopentanoate (see Fig. 3) as the main component (Randová et al. 2016). Consideration of this compound as a dipolar aprotic solvent, to replace materials such as N*-methylpyrrolidone* in some synthesis applications, has led Cseri and Szekely (2019)) to investigate alternative routes to purer material. The best of these, from methyl propionate and *N,N*-diethylacrylamide, yielded material of > 99% purity. A comparison was also made of the overall carbon intensities of the various routes. These provide a measure of the quantity of waste each route produces per kg of product. However, all the routes currently use fossil-carbon sourced raw materials rather than renewable.
