# Supplementary material for: The green solvent: a critical perspective
Source: Clean Technol Environ Policy. 2021 Sep 30;23(9):2499–522. doi: 10.1007/s10098-021-02188-8 (PMC8482956; doi:10.1007/s10098-021-02188-8)
Supplement: Supplementary file 1 — Supplementary file1 (DOCX 63 kb) [file 10098_2021_2188_MOESM1_ESM.docx]

Supplementary Information for:

Clean Technologies and Environmental Policy

**The Green Solvent – a Critical Perspective**

Neil Winterton, Department of Chemistry,

University of Liverpool, Liverpool, L69 7ZD, UK.

[n.winterton@liverpool.ac.uk](mailto:n.winterton@liverpool.ac.uk) (ORCID 0000-0002-3887-4198)

**ONLINE RESOURCE 1 Table S1 Classifications of Solvents Listed in Various Solvent Selection Guides**

The solvents listed come from a range of solvent types, classified according to their composition (aliphatic or aromatic hydrocarbon, oxygenated, halogenated), the presence of one or more functional groups (alcohol, ester, ether, ketone, carboxylic acid, amine, sulfoxide, sulfone, phosphoramide) or their molecular, physical or chemical characteristics relevant to solvent property or behaviour (such as non-polar, dipolar, polar aprotic, polar, acidic, basic, hydrogen-bond donor or acceptor) and various assessments of safety, health and environmental impact. All those listed in Table S1 are long-known chemical species. Indeed, according to data accessed *via* their CAS Registry Number (RN)^[[1]](#footnote-1)^ most have been known for more than 100 years. This is hardly surprising as the compounds listed in the Selection Guides are there because they are used in conventional chemical synthesis and in industrial chemicals manufacture to meet contemporary techno-commercial criteria, such as price, assured availability and regulatory approval for use.

None of the Guides, therefore, is designed to guide the search for new solvents. However, more recently, Astra Zeneca (Diorazio et al 2016) and Syngenta (Piccione et al 2019) have reported computer-based interactive selection tools which complement the Selection Guides, including additional solvents in the data sets.

Solvent Selection Guides (Prat et al 2013; Alder et al 2016; Prat et al 2016; Alfonsi et al 2008; http://www.acs.org/content/dam/acsorg/greenchemistry/industriainnovation/roundtable/acs-gcipr-solvent-selection-guide.pdf) provide overall empirical assessments, expressed numerically or graphically, of a combination of critical safety, health and environmental (SHE) factors, such as toxicity (chronic, acute, carcinogenicity, teratogenicity), safety in use (flammability, explosion hazard, corrosivity) and environmental impact (ecotoxicity, ozone depletion potential, photochemical ozone creation potential, greenhouse warming potential, persistence). The guides use a ‘traffic light’ system to categorise individual solvents, with each colour representing a different descriptor, as follows:

*Green:* ‘recommended’ (Prat et al 2013), ‘few known issues’ (Alder et al 2016), ‘recommended’ (Prat

et al 2016) and ‘preferred’ (Alfonsi et al 2008);

*Amber:* ‘substitution advisable’ (Prat et al 2013), ‘some known issues’ (Alder et al 2016), ‘problematic’

(Prat et al 2016) and ‘usable’ (Alfonsi et al 2008);

*Red*: ‘substitution requested’, (Prat et al 2013), ‘major known issues’ (Alder et al 2016), ‘hazardous’

(Prat et al 2016) and ‘undesirable’ (Alfonsi et al 2008).

One of the guides includes a fourth classification: ‘highly hazardous’ (Prat et al 2016).

The different scoring methods for particular criteria and the different weighting given in the aggregation of scores give rise to some differences in the final colour-coded categorisations applied to particular solvents.

There is, however, broad agreement (Tobiszewski et al 2015) between the Guides regarding those chemical types considered of limited hazard and those best avoided. They provide a useful starting point for academic and fundamental studies searching for alternatives and replacements for specific compounds, such as N,N-*dimethylformamide*, and compound types, such as dipolar aprotics. For researchers, they are convenient sources of information to supplement the knowledge and chemical intuition of the practitioner in that they highlight the range of factors likely to determine solvent impact in general. They also highlight important gaps in the extent of, and limitations to the quality of, information for many solvents, particularly relating to toxicology and ecotoxicology.^[[2]](#footnote-2)^ This has given rise to publications (Łuczyńska et al 2018) seeking to use computational methods to fill in the gaps. Such methods clearly need some independent validation and verification before they can be used for anything but speculative investigations.

The rankings for the solvents included in the industrial Selection Guides have been compared (Tobiszewski et al 2015) with the physicochemical data for 151 other solvents, subjected to cluster analysis and then ranked according to hazard-related data. Confidence levels were assigned to these rankings based on the availability or lack of such hazard-related data.

**REFERENCES**

Alder CM, Hayler JD, Henderson RK, Redman AM, Shukla L, Shuster LE and Sneddon HF (2016) Updating and further expanding GSK’s solvent sustainability guide. Green Chem. **18:**3879-3890

Alfonsi K, Colberg J, Dunn PJ, Fevig T, Jennings S, Johnson TA, Kleine HP, Knight C, Nagy MA, Perry DA and Stefaniak M (2008) Green chemistry tools to influence medicinal chemistry and research chemistry based organisation. Green Chem **10:**31-36

Diorazio LJ, Hose DRJ and Adlington NK (2016) Towards a More Holistic Framework for Solvent Selection. Org Process Res Dev **20:**760-773

Łuczyńska, G. Pena-Pereira F, Tobiszewski M and Namieśnik J (2018) Expectation Maximisation Model for Substitution of Missing Values Characterizing Greenness of Organic Solvents. Molecules **23:**1292

Piccione PM, Baumeister J, Salvesen T, Grosjean C, Flores Y, Groelly E, Murudi V, Shyadligeri A, Lobanova O and Lothschütz C (2019) Solvent Selection Method and Tool. Org Process Res Dev. **23:**998-1016

Prat D, Pardigon O, Flemming H-W, Letestu S, Ducandas V, Isnard P, Guntrum E, Senac T, Ruisseau S, Cruciani P and Hosek PK (2013) Sanofi’s Solvent Selection Guide: A Step Toward More Sustainable Processes. Org Process Res Dev **17:**1517-1525

Prat D, Hayler J and Wells A (2014) A survey of solvent selection guides. Green Chem 16:4546-4551

Prat D, Wells A, Hayler J, Sneddon H, McElroy CR, Abou-Shehada S and Dunn PJ (2016) CHEM21 selection guide of classical- and less classical-solvents. Green Chem **18:**288-296

Tobiszewski M, Tsakovski S, Simeonov V, Namieśnika J and Pena-Pereira F (2015) A solvent selection guide based on chemometrics and multicriteria decision analysis. Green Chem **17:**4773-4785 (and correction: Green Chem 17:5206)

|  | **TABLE S1** | **CLASSIFICATIONS OF SOLVENT LISTED IN VARIOUS SELECTION GUIDES^(g,h)^** | | | | | | | | | | | | | | | |  | |  |
| --- | --- | --- | --- | --- | --- | --- | --- | --- | --- | --- | --- | --- | --- | --- | --- | --- | --- | --- | --- | --- |
|  |  |  |  |  |  | | |  | |  |  | | |  | |  | | |  |  |
| Entry | **SOLVENT** | CHEM21 | GSK | Sanofi | GCI-PR | | | Pfizer | | Astra Zeneca | CAS Registry | | | Molecular | | Boiing Point^(i)^ | | |  |  |
| number |  | 2016^(a)^ | 2016^(b)^ | 2013^(c)^ | 2011^(d)^ | | | 2008^(e)^ | | 2008^(f)^ | Number | | | Formula | | (°C) | | |  |  |
|  |  | Rankings | Composite colour | Rankings |  | | | Composite colour | | Composite colour |  | | |  | |  | | |  |  |
|  | **ACIDS/ANHYDRIDE** |  |  |  |  | | |  | |  |  | | |  | |  | | |  |  |
| 1 | formic acid | P/P* | Amber | Red | Included | | |  | | Amber | 64-18-6 | | | CH2O2 | | 100.5 | | |  |  |
| 2 | trifluoromethanesulfonic acid |  |  | Red |  | | |  | |  | 1493-13-6 | | | CHF3O3S | | 166 | | |  |  |
| 3 | methanesulfonic acid |  | Amber | Red |  | | |  | |  | 75-75-2 | | | CH4O3S | | 167/10 torr | | |  |  |
| 4 | trifluoroacetic acid |  | Red | Amber |  | | |  | |  | 76-05-1 | | | C2HF3O2 | | 73 | | |  |  |
| 5 | acetic acid | P/P* | Amber | Amber | Included | | | Amber | | Green | 64-19-7 | | | C2H4O2 | | 117.9 | | |  |  |
| 6 | propionic acid |  | Green | Amber | Included | | |  | |  | 79-09-4 | | | C3H6O2 | | 141.1 | | |  |  |
| 7 | lactic acid | P | Green |  |  | | |  | |  | 50-21-5 | | | C3H6O3 | | 122/14-15 torr | | |  |  |
|  |  |  |  |  |  | | |  | |  |  | | |  | |  | | |  |  |
| 8 | acetic anhydride | P/P* | Amber | Amber | Included | | |  | |  | 108-24-7 | | | C4H6O3 | | 139.5 | | |  |  |
|  |  |  |  |  |  | | |  | |  |  | | |  | |  | | |  |  |
|  | **ALCOHOLS/DIOLS/TRIOL** |  |  |  |  | | |  | |  |  | | |  | |  | | |  |  |
| 9 | methanol | P/R* | Amber | Green | Included | | | Green | | Amber | 67-56-1 | | | CH4O | | 64.7 | | |  |  |
| 10 | ethanol | R/R* | Green | Green | Included | | | Green | | Green | 64-17-5 | | | C2H6O | | 78.5 | | |  |  |
| 11 | 1-propanol |  | Green | Green | Included | | | Green | |  | 71-23-8 | | | C3H8O | |  | | |  |  |
| 12 | 2-propanol | R/R* | Green | Green | Included | | | Green | | Green | 67-63-0 | | | C3H8O | | 82.5 | | |  |  |
| 13 | 1-butanol | R/R* | Green | Green | Included | | | Green | | Greem | 71-36-3 | | | C4H10O | | 117.7 | | |  |  |
| 14 | *iso*-butanol | R | Green | Green | Included | | |  | |  | 78-83-1 | | | C4H10O | | 108 | | |  |  |
| 15 | 2-butanol |  |  | Green | Included | | | Green | |  | 78-92-2 | | | C4H10O | | 99.5 | | |  |  |
| 16 | *tert*-butanol | R/R* | Amber | Amber | Included | | |  | | Amber | 75-65-0 | | | C4H10O | | 82.4 (mp 25.7) | | |  |  |
| 17 | 1-pentanol |  | Green |  |  | | |  | |  | 71-41-0 | | | C5H12O | | 137.5 | | |  |  |
| 18 | *iso*-amyl alcohol | R |  |  | Included | | |  | |  | 123-51-3 | | | C5H12O | | 132.5 | | |  |  |
| 19 | *tert*-amyl alcohol |  | Green | Amber |  | | |  | |  | 75-85-4 | | | C5H12O | | 102.4 | | |  |  |
| 20 | 1-hexanol |  | Green |  |  | | |  | |  | 111-27-3 | | | C6H14O | | 157 | | |  |  |
| 21 | benzyl alcohol | P/P* |  | Amber | Included | | |  | |  | 100-51-6 | | | C7H8O | | 205.3 | | |  |  |
| 22 | cyclohexanol |  |  | Amber |  | | |  | |  | 108-93-0 | | | C7H14O | | 161.1 | | |  |  |
| 23 | 1-heptanol |  | Green |  |  | | |  | |  | 111-70-6 | | | C7H16O | | 175.8 | | |  |  |
| 24 | 1-octanol |  | Green |  |  | | |  | |  | 111-87-5 | | | C8H18O | | 194-195 | | |  |  |
|  |  |  |  |  |  | | |  | |  |  | | |  | |  | | |  |  |
| 25 | ethylene glycol | R/R* | Green | Amber | Included | | | Amber | |  | 107-21-1 | | | C2H6O2 | | 197.3 | | |  |  |
| 26 | 1,2-propanediol |  |  | Amber |  | | |  | |  | 57-55-6 | | | C3H8O2 | | 188.2 | | |  |  |
| 27 | 1,3-propanediol | P |  | Amber |  | | |  | |  | 504-63-2 | | | C3H8O2 | | 214.4 | | |  |  |
| 28 | 1,4-butanediol |  | Green |  |  | | |  | |  | 110-63-4 | | | C4H10O2 | | 230 | | |  |  |
| 29 | glycerol | P |  |  |  | | |  | |  | 56-81-5 | | | C3H8O3 | | 290 | | |  |  |
|  |  |  |  |  |  | | |  | |  |  | | |  | |  | | |  |  |
|  | **KETONES/ALDEHYDE** |  |  |  |  | | |  | |  |  | | |  | |  | | |  |  |
| 30 | acetone | P/R* | Amber | Green | Included | | | Green | | Amber | 67-64-1 | | | C3H6O | | 56.0 | | |  |  |
| 31 | methyl ethyl ketone | R/R* | Amber | Green | Included | | | Green | | Amber | 78-93-3 | | | C4H8O | | 79.6 | | |  |  |
| 32 | cyclopentanone |  | Green | Amber |  | | |  | |  | 120-92-3 | | | C5H8O | | 130.6 | | |  |  |
| 33 | 3-pentanone |  | Green |  |  | | |  | |  | 96-22-0 | | | C5H10O | | 101.9 | | |  |  |
| 34 | methyl isobutyl ketone | R/R* | Green | Green | Included | | |  | | Amber | 108-10-1 | | | C6H12O | | 115.8 | | |  |  |
| 35 | 2-hexanone |  |  | Red |  | | |  | |  | 591-78-6 | | | C6H12O | | 127.6 | | |  |  |
| 36 | cyclohexanone | R/P* |  | Amber | Included | | |  | |  | 108-94-1 | | | C6H10O | | 156 | | |  |  |
|  |  |  |  |  |  | | |  | |  |  | | |  | |  | | |  |  |
| 37 | furfuraldehyde |  | Green |  |  | | |  | |  | 98-01-1 | | | C5H4O2 | | 162 | | |  |  |
|  |  |  |  |  |  | | |  | |  |  | | |  | |  | | |  |  |
|  | **ESTERS** |  |  |  |  | | |  | |  |  | | |  | |  | | |  |  |
| 38 | methyl formate |  | Red |  | Included | | |  | |  | 107-31-3 | | | C2H4O2 | | 31.5 | | |  |  |
| 39 | ethyl formate |  |  | Amber |  | | |  | |  | 109-94-4 | | | C3H6O2 | | 54.5 | | |  |  |
| 40 | methyl acetate | P/P* |  | Amber | Included | | |  | |  | 79-20-9 | | | C3H6O2 | | 98 | | |  |  |
| 41 | ethyl acetate | R/R* | Green | Green | Included | | | Green | | Green | 141-78-6 | | | C4H8O2 | | 77 | | |  |  |
| 42 | methyl propionate |  | Amber |  |  | | |  | |  | 554-12-1 | | | C4H8O2 | | 79.8 | | |  |  |
| 43 | n-propyl acetate |  |  | Green |  | | |  | |  | 109-60-4 | | | C5H10O2 | | 101.5 | | |  |  |
| 44 | *iso*-propyl acetate | R/R* | Green | Green | Included | | | Green | | Green | 108-21-4 | | | C5H10O2 | | 88.6 | | |  |  |
| 45 | ethyl propionate |  | Amber |  |  | | |  | |  | 105-37-3 | | | C5H10O2 | | 90.2 | | |  |  |
| 46 | *n*-butyl acetate | R/R* |  | Green | Included | | |  | | Green | 123-86-4 | | | C6H12O2 | | 126.1 | | |  |  |
| 47 | *iso*-butyl acetate | R | Green | Amber | Included | | |  | |  | 110-19-0 | | | C6H12O2 | | 116.5 | | |  |  |
| 48 | *tert*-butyl acetate |  | Amber |  |  | | |  | |  | 540-88-5 | | | C6H12O2 | | 97.8 | | |  |  |
| 49 | amyl acetate |  | Green |  | Included | | |  | |  | 628-63-7 | | | C7H14O2 | | 149.2 | | |  |  |
| 50 | *iso*-amyl acetate | R | Green |  |  | | |  | |  | 123-92-2 | | | C7H14O2 | | 142.5 | | |  |  |
| 51 | 2-ethylhexyl acetate |  | Green |  |  | | |  | |  | 103-09-3 | | | C10H20O2 | | 199 | | |  |  |
| 52 | methyl oleate |  | Green |  |  | | |  | |  | 112-62-9 | | | C19H36O2 | | 218.5/20 torr | | |  |  |
| 53 | ethyl lactate | P |  | Amber |  | | |  | |  | 97-64-3 | | | C5H1003 | | 154 | | |  |  |
|  |  |  |  |  |  | | |  | |  |  | | |  | |  | | |  |  |
| 54 | dimethyl adipate |  | Amber |  |  | | |  | |  | 627-93-0 | | | C8H14O4 | | 115/13 torr | | |  |  |
| 55 | dimethyl succinate |  | Green |  |  | | |  | |  | 106-65-0 | | | C6H10O4 | | 195.3 | | |  |  |
| 56 | diethyl succinate | P | Amber |  |  | | |  | |  | 123-25-1 | | | C8H14O4 | | 217.7 | | |  |  |
| 57 | di-*iso*-propyl adipate |  | Amber |  |  | | |  | |  | 6938-94-9 | | | C12H22O4 | | 113-115/.5 torr | | |  |  |
| 58 | glycol diacetate | R | Green | Amber |  | | |  | |  | 111-55-7 | | | C6H10O4 | | 190-191 | | |  |  |
| 59 | glycerol triacetate |  | Green | Amber |  | | |  | |  | 102-76-1 | | | C9H14O6 | | 258-260 | | |  |  |
|  |  |  |  |  |  | | |  | |  |  | | |  | |  | | |  |  |
| 60 | γ-butyrolactone |  |  | Red |  | | |  | |  | 96-48-0 | | | C4H6O2 | | 204 | | |  |  |
| 61 | γ-valerolactone | P | Amber | Amber |  | | |  | |  | 108-29- 2 | | | C5H8O2 | | 85 | | |  |  |
|  |  |  |  |  |  | | |  | |  |  | | |  | |  | | |  |  |
| 62 | ethylene carbonate | P |  |  |  | | |  | |  | 96-49-1 | | | C3H4O3 | | 248 | | |  |  |
| 63 | dimethyl carbonate | R | Green |  | Included | | |  | |  | 616-38-6 | | | C3H6O3 | | 90-91 | | |  |  |
| 64 | propyene carbonate | P | Green |  |  | | |  | |  | 108-32-7 | | | C4H8O3 | | 242 | | |  |  |
| 65 | butylene carbonate |  | Amber |  |  | | |  | |  | 4437-85-8 | | | C5H8O3 | | 251 | | |  |  |
| 66 | diethyl carbonate |  | Green |  |  | | |  | |  | 105-58-8 | | | C5H10O3 | | 126 | | |  |  |
|  |  |  |  |  |  | | |  | |  |  | | |  | |  | | |  |  |
|  | **ETHERS** |  |  |  |  | | |  | |  |  | | |  | |  | | |  |  |
| 67 | tetrahydrofuran | P/P* | Red | Amber | Included | | | Amber | | Red | 109-99-9 | | | C4H8O | | 65 | | |  |  |
| 68 | diethyl ether | H/HH* | Red | Brown | Included | | | Red | | Red | 60-29-7 | | | C4H10O | | 35 | | |  |  |
| 69 | 2-methyltetrahydrofuran | P/P* | Amber | Green | Included | | | Amber | | Red | 96-47-9 | | | C5H10O | | 78 | | |  |  |
| 70 | *tert*-butyl methyl ether | H/H* | Red | Amber | Included | | | Amber | | Red | 1634-04-4 | | | C5H12O | | 55.2 | | |  |  |
| 71 | cyclopentyl methyl ether | P | Amber | Red | Included | | |  | |  | 5614-37-9 | | | C6H12O | | 105.4 | | |  |  |
| 72 | di-*iso*-propyl ether | H/H* | Red | Amber |  | | | Red | |  | 108-20-3 | | | C6H14O | | 68.5 | | |  |  |
| 73 | ethyl *tert*-butyl ether | P |  | Red |  | | |  | |  | 637-92-3 | | | C6H14O | | 73.1 | | |  |  |
| 74 | *tert*-amyl methyl ether | R |  |  |  | | |  | |  | 994-05-8 | | | C6H14O | | 86.3 | | |  |  |
| 75 | anisole | P/R* | Green | Green | Included | | |  | | Green | 100-66-3 | | | C7H8O | | 155.5 | | |  |  |
| 76 | ethoxybenzene |  | Green |  |  | | |  | |  | 103-73-1 | | | C8H10O | | 171-173 | | |  |  |
| 77 | dibutyl ether |  |  | Amber |  | | |  | |  | 142-96-1 | | | C8H18O | | 140.2 | | |  |  |
| 78 | diphenyl ether |  | Amber |  |  | | |  | |  | 101-84-8 | | | C12H10O | | 259 | | |  |  |
|  |  |  |  |  |  | | |  | |  |  | | |  | |  | | |  |  |
| 79 | 1,4-dioxan | P/H* | Red | Red | Included | | | Red | | Red | 123-91-1 | | | C4H8O2 | | 101.1 | | |  |  |
| 80 | 1,3-dioxolane |  | Amber |  |  | | |  | |  | 646-06-0 | | | C3H6O2 | | 78 | | |  |  |
| 81 | 1,2-dimethoxyethane | H/H* | Red | Red | Included | | | Red | | Amber | 110-71-4 | | | C4H10O2 | | 82-83 | | |  |  |
| 82 | dimethoxymethane |  | Red |  |  | | |  | |  | 109-87-5 | | | C3H8O2 | | 41.6 | | |  |  |
| 83 | ethyleneglycol monomethyl ether | H/H* |  | Red | Included | | |  | | Amber | 109-86-4 | | | C3H8O2 | | 124.1 | | |  |  |
| 84 | ethyleneglycol monoethyl ether |  |  | Red |  | | |  | |  | 110-80-5 | | | C4H10O2 | | 135.6 | | |  |  |
| 85 | 1-methoxy-2-propanol |  |  | Amber |  | | |  | |  | 107-98-2 | | | C4H10O2 | | 120.1 | | |  |  |
| 86 | furfuryl alcohol |  |  | Amber |  | | |  | |  | 98-00-0 | | | C5H6O2 | | 171 | | |  |  |
| 87 | tetrahydrofurfuryl alcohol | H | Red | Amber |  | | |  | |  | 97-99-4 | | | C5H10O2 | | 178 | | |  |  |
| 88 | diethoxymethane |  | Amber | Red |  | | |  | |  | 462-95-3 | | | C5H12O2 | | 88 | | |  |  |
|  |  |  |  |  |  | | |  | |  |  | | |  | |  | | |  |  |
| 89 | diethyleneglycol |  | Green |  |  | | |  | |  | 111-46-6 | | | C4H10O3 | | 244-245 | | |  |  |
| 90 | diethyleneglycol dimethyl ether |  |  | Red | Included | | |  | |  | 111-96-6 | | | C6H14O3 | | 162 | | |  |  |
| 91 | 1,2,3-trimethoxypropane |  | Amber |  |  | | |  | |  | 20637-49-4 | | | C6H14O3 | | 148 | | |  |  |
| 92 | diethylene glycol monobutyl ether |  | Green |  |  | | |  | |  | 112-34-5 | | | C8H18O3 | | 230.4 | | |  |  |
| 93 | dihydrolevoglucosenone | P | Amber |  |  | | |  | |  | 53716-82-8 | | | C6H8O3 | | 231.6±30.0 (est) | | |  |  |
| 94 | 2,2-dimethyl-1,3-dioxolane-4-methanol |  | Amber |  |  | | |  | |  | 100-79-8 | | | C6H12O3 | | 188 | | |  |  |
| 95 | triethyleneglycol |  | Green |  |  | | |  | |  | 112-27-6 | | | C6H14O4 | | 285 | | |  |  |
| 96 | dimethyl *iso*-sorbide |  | Amber |  |  | | |  | |  | 5306-85-4 | | | C8H14O4 | | 234 | | |  |  |
|  |  |  |  |  |  | | |  | |  |  | | |  | |  | | |  |  |
|  | **HYDROCARBONS** |  |  |  |  | | |  | |  |  | | |  | |  | | |  |  |
| 97 | pentane | H/H* |  | Brown |  | | | Red | |  | 109-66-0 | | | C5H12 | | 36.1 | | |  |  |
| 98 | hexane | H/H* | Red | Red | Included | | | Red | | Red | 110-54-3 | | | C6H14 | | 68.7 | | |  |  |
| 99 | heptane | P/P* | Amber | Amber | Included | | | Amber | | Amber | 142-82-5 | | | C7H16 | | 98 | | |  |  |
| 100 | cyclohexane | P/P* | Amber | Amber | Included | | | Amber | | Red | 110-82-7 | | | C6H12 | | 80,7 | | |  |  |
| 101 | methylcyclohexane | P/P* |  | Amber | Included | | | Amber | |  | 108-87-2 | | | C7H14 | | 100.9 | | |  |  |
| 102 | octane |  |  | Red |  | | |  | |  | 111-65-9 | | | C8H18 | | 125.7 | | |  |  |
| 103 | *iso*-octane |  | Amber |  | Included | | | Amber | |  | 540-84-1 | | | C8H18 | | 99.2 | | |  |  |
|  |  |  |  |  |  | | |  | |  |  | | |  | |  | | |  |  |
| 104 | benzene | H/HH* | Red | Brown | Included | | | Red | |  | 71-43-2 | | | C6H6 | | 80.1 | | |  |  |
| 105 | toluene | P/P* | Amber | Amber | Included | | | Amber | | Amber | 108-88-3 | | | C7H8 | | 110.6 | | |  |  |
| 106 | xylene(s) | P/P* |  | Amber | Included | | | Amber | | Amber |  | | | C8H10 | |  | | |  |  |
| 106a | p-xylene |  | Amber |  |  | | |  | |  | 106-42-3 | | | C8H10 | | 138.4 | | |  |  |
| 107 | cumene |  |  | Amber |  | | |  | |  | 98-82-8 | | | C9H12 | | 152.4 | | |  |  |
| 108 | mesitylene |  | Amber |  |  | | |  | |  | 108-67-8 | | | C9H12 | | 164.7 | | |  |  |
| 109 | 1,2,3,4-tetrahydronaphthalene |  |  | Red |  | | |  | |  | 119-64-2 | | | C10H12 | | 207.6 | | |  |  |
| 110 | *p*-cymene | P | Amber |  |  | | |  | |  | 99-87-6 | | | C10H14 | | 177.1 | | |  |  |
| 111 | limonene |  |  | Amber |  | | |  | |  | 138-86-3 | | | C10H16 | | 176 | | |  |  |
| 111a | L-limonene |  | Amber |  |  | | |  | |  | 5989-54-8 | | | C10H16 | | 176 | | |  |  |
| 111b | D-limonene | P | Amber |  |  | | |  | |  | 5989-27-5 | | | C10H16 | | 176 | | |  |  |
| 112 | *cis*-decalin |  | Amber |  |  | | |  | |  | 493-01-6 | | | C10H18 | | 195.8 | | |  |  |
|  |  |  |  |  |  | | |  | |  |  | | |  | |  | | |  |  |
|  | **WITH HETEROATOMS OTHER THAN OXYGEN** | |  | | |  |  | |  | | |  |  | |  | |  | | | |
| 113 | acetonitrile | R/P* | Amber | Green | Included | | | Amber | | Red | 75-05-8 | | | C2H3N | | 81.6 | | |  |  |
| 114 | propionitrile |  |  | Red |  | | |  | |  | 107-12-0 | | | C3H5N | | 97.2 | | |  |  |
| 115 | pyridine | R/H* | Red | Amber | Included | | | Red | | Red | 110-86-1 | | | C5H5N | | 115.2 | | |  |  |
| 116 | triethylamine | P/H* |  | Red | Included | | |  | | Red | 121-44-8 | | | C6H15N | | 89.3 | | |  |  |
| 117 | 2,4,6-collidine |  | Green |  |  | | |  | |  | 108-75-8 | | | C8H11N | | 170.4 | | |  |  |
| 118 | *N,N*-dimethylaniline |  | Amber |  |  | | |  | |  | 121-69-7 | | | C8H11N | | 193.5 | | |  |  |
| 119 | *N*-methylformide |  | Red |  |  | | |  | |  | 123-39-7 | | | C2H5NO | | 180-185 | | |  |  |
| 120 | *N,N*-dimethylformamide | H/H* | Red | Red | Included | | | Red | | Amber | 68-12-2 | | | C3H7NO | | 153 | | |  |  |
| 121 | *N,N*-dimethylacetamide | H/H* | Red | Red | Included | | |  | | Amber | 127-19-5 | | | C4H9NO | | 163-165 | | |  |  |
| 122 | *N*-methyl-2-pyrrolidone | H/H* | Red | Red | Included | | | Red | | Green | 872-50-4 | | | C5H9NO | | 202 | | |  |  |
| 123 | *N*-ethyl-2-pyrrolidone |  | Red |  |  | | |  | |  | 2687-91-4 | | | C6H11NO | | 218 | | |  |  |
| 124 | *N,N*-dimethyloctanamide |  | Amber |  |  | | |  | |  | 1118-92-9 | | | C10H21NO | | 175-177/0.15 torr | | |  |  |
| 125 | *N,N*-dimethyldecanamide |  | Amber |  |  | | |  | |  | 14433-76-2 | | | C12H25NO | | 152/12 torr | | |  |  |
| 126 | nitromethane | H/HH* | Amber | Brown |  | | |  | |  | 75-52-5 | | | CH3NO2 | | 101.2 | | |  |  |
| 127 | tetramethylurea |  | Red |  | Included | | |  | |  | 632-22-4 | | | C5H12N2O | | 176.5 | | |  |  |
| 128 | 1,3-dimethylethylene urea |  | Amber | Amber | Included | | |  | |  | 80-73-9 | | | C5H10N2O | | 221 | | |  |  |
| 129 | 1,3-dimethylpropylene urea | P/P* |  | Amber |  | | |  | |  | 7226-23-5 | | | C6H12N2O | | 164/44 torr | | |  |  |
|  |  |  |  |  |  | | |  | |  |  | | |  | |  | | |  |  |
| 130 | dimethyl sulfoxide | R/P* | Amber | Amber | Included | | | Amber | | Green | 67-68-5 | | | C2H6OS | | 189 | | |  |  |
| 131 | sulfolane | H/H* | Red | Amber | Included | | |  | | Green | 126-33-0 | | | C4H8O2S | | 285 | | |  |  |
| 132 | hexamethylphosphoramide | H/HH* |  |  |  | | |  | |  | 680-31-9 | | | C6H18N3OP | | 233 | | |  |  |
| 133 | carbon disulfide | H/HH* | Red |  |  | | |  | |  | 75-15-0 | | | CS2 | | 45 | | |  |  |
| 134 | dichloromethane | H/H* | Red | Amber | Included | | | Red | | Amber | 75-09-2 | | | CH2Cl2 | | 40 | | |  |  |
| 135 | chloroform | P/HH* | Red | Brown | Included | | | Red | |  | 67-66-3 | | | CHCl3 | | 61.2 | | |  |  |
| 136 | carbon tetrachloride | H/HH* | Red | Brown | Included | | | Red | |  | 56-23-5 | | | CCl4 | | 76.8 | | |  |  |
| 137 | *cis*-dichloroethylene |  |  | Red |  | | |  | |  | 156-59-2 | | | C2H2Cl2 | | 60.1 | | |  |  |
| 138 | *trans*-dichloroethylene |  |  | Red |  | | |  | |  | 156-60-5 | | | C2H2Cl2 | | 48.7 | | |  |  |
| 139 | 1,2-dichloroethane | H/HH* | Red | Brown | Included | | | Red | |  | 107-06-2 | | | C2H4Cl2 | | 83.5 | | |  |  |
| 140 | 1,1,1-trichloroethane |  |  | Brown |  | | |  | |  | 71-55-6 | | | C2H3Cl3 | | 74 | | |  |  |
| 141 | 1,1,2-trichloroethane |  |  | Brown |  | | |  | |  | 79-00-5 | | | C2H3Cl3 | | 113.8 | | |  |  |
| 142 | trichloroacetonitrile | R |  |  |  | | |  | |  | 545-06-2 | | | C2Cl2N | | 85.7 | | |  |  |
| 143 | chlorobenzene | P/P* |  | Amber | Included | | |  | | Red | 108-90-7 | | | C6H5Cl | | 131.7 | | |  |  |
| 144 | 1,2-dichlorobenzene |  |  | Red |  | | |  | |  | 95-50-1 | | | C6H4Cl2 | | 180.1 | | |  |  |
| 145 | fluorobenzene |  | Amber |  |  | | |  | |  | 462-06-6 | | | C6H5F | | 84.7 | | |  |  |
| 146 | tetradecafluorohexane |  | Amber |  |  | | |  | |  | 355-42-0 | | | C6F14 | | 56.6 | | |  |  |
| 147 | benzotrifluoride |  | Amber |  | Included | | |  | |  | 98-08-8 | | | C7H5F3 | | 103,5 | | |  |  |
| 148 | octafluorotoluene |  | Green |  |  | | |  | |  | 434-64-0 | | | C7F8 | | 104.5 | | |  |  |
| 149 | 2,2,2-trifluoroethanol |  | Red |  |  | | |  | |  | 75-89-8 | | | C2H3F3O | | 74 | | |  |  |
| 150 | perfluoro-2-butyltetrahydrofuran |  | Amber |  |  | | |  | |  | 335-36-4 | | | C8F16O | | 102.6 | | |  |  |
|  |  |  |  |  |  | | |  | |  |  | | |  | |  | | |  |  |
|  | **BLENDS or MIXTURES** |  |  |  |  | | |  | |  |  | | |  | |  | | |  |  |
| 151 | industrial methylated spirits |  | Amber |  |  | | |  | |  | 64-17-5 | | | for EtOH | |  | | |  |  |
| 152 | petroleum spirits |  | Red |  |  | | |  | |  | 8032-32-4 | | |  | | 60-110 | | |  |  |
| 153 | turpentine | P |  | Amber |  | | |  | |  | 9005-90-7 | | |  | |  | | |  |  |
|  |  |  |  |  |  | | |  | |  |  | | |  | |  | | |  |  |
|  | **INORGANIC** |  |  |  |  | | |  | |  |  | | |  | |  | | |  |  |
| 154 | water | R/R* | Green | Green |  | | | Green | |  | 7732-18-5 | | | H2O | | 100 | | |  |  |

Footnotes:

^a^ Prat et al (2016). Ranking by cumulative score (R/P/H/HH) and additional ranking following discussion (R*/P*/H*/HH*): R = Recommended; P = Problematic; H = Hazardous; HH = Highly Hazardous

^b^ Alder et al (2016). Colour ranking based on cumulative score: Green/Amber/Red.

^c^ Prat et al (2013). Overall ranking: Green (recommended)/Amber (Substitution advisable)/Red (Substitution recommended)/Brown (Banned).

^d^ GCI-PR (2011). <http://www.acs.org/content/dam/acsorg/greenchemistry/industriainnovation/roundtable/acs-gcipr-solvent-selection-guide.pdf>. Selected for inclusion in the ACS GCI Pharmaceuticals Roundtable Guide with individual safety, health and environment (air, water and waste) scores, where data are available.

^e^ Alfonsi et al (2008). Classified according to a colour code: Green (Preferred)/Amber (Usable)/Red (Undesirable).

^f^ Astra Zeneca (2008). Rankings taken from Prat D, Hayler J and Wells A (2014) whose ref. 10 notes the following: ‘AZ guide has not been published but was presented in the Green Chemistry Institute Pharmaceutical Roundtable in 2008. See document entitled ‘Collaboration to deliver a solvent selection guide for the pharmaceutical industry.’ by C. R. Hargeaves and J. B. Manley on GCI-PR website, ref. 2.’ The original AZ guide consists of 46 solvents assessed using 10 criteria aggregated and assigned a green/amber/red colour code. The colour codes are taken from Table 1 of Prat D, Hayler and Wells A (2014).

^g^ This table is provided for information only. Inclusion in any of the guides is not a recommendation for use or non-use. Please see original references to understand the methodologies used and the data in which the selection was based, bearing in mind that updated information will have become available since publication.

^h^ Solvents grouped into main chemical/functional group classes and then according to molecular formula C_t_H_u_X_v_N_w_O_x_P_y_S_z_, where X = Cl, F.

^I^ Representative boiling points are taken from the individual CAS Registry Number record at either atmospheric or reduced pressure. Where no boiling point measurement has been reported an estimated boiling point provided in the CAS RN entry is given.

1. The Chemical Abstracts Service Registry Number (CAS RN) is a unique identifier for chemical substances the characteristics of which have been published and abstracted by the Chemical Abstracts Service of the American Chemical Society. The use of this number as a search term in the SciFinder^®^ data base (http://www.scifinder-n.cas.org) provides links to associated chemical literature, key physical and chemical properties and other useful information. The unique identifier is particularly useful when an individual compound has a number of common, technical and scientific names. Where known, CAS RNs are listed in all the compound tabulations in the main text. [↑](#footnote-ref-1)
2. A list of the toxicological and ecological characteristics of solvents for which credible and reliable data are needed was published in 2009 by the US Environmental Protection Agency (epa.gov/sites/production/files/2014-02/documents/dfe_screen_for_solvents-in-cleaning-products-february2009.pdf). [↑](#footnote-ref-2)
