# Supplementary material for: The green solvent: a critical perspective
Source: Clean Technol Environ Policy. 2021 Sep 30;23(9):2499–522. doi: 10.1007/s10098-021-02188-8 (PMC8482956; doi:10.1007/s10098-021-02188-8)
Supplement: Supplementary file 2 — Supplementary file2 (DOCX 108 kb) [file 10098_2021_2188_MOESM2_ESM.docx]

Supplementary Information for:

Clean Technologies and Environmental Policy

**The Green Solvent – a Critical Perspective**

Neil Winterton, Department of Chemistry,

University of Liverpool, Liverpool, L69 7ZD, UK.

[n.winterton@liverpool.ac.uk](mailto:n.winterton@liverpool.ac.uk) (ORCID 0000-0002-3887-4198)

**ONLINE RESOURCE 2 TABLE S2 Additional Solvents listed in Various Compilations and Papers**

Table S2 displays a set of 425 materials compiled from a series published studies devoted to the search for new solvents. Most of the materials listed are unique chemical species. Also included is a small number of mixtures, some of specified and some of variable or of ill-defined composition, as well as one or two technical grade commercial formulations of unspecified composition. The great majority have CAS Registry Numbers (CAS RN).^(a)^ None of the materials is one of the 154 compounds in Table S1 from Solvent Selection Guides.

Table S2 contains only a small number of ionic liquids, specifically only those included in general solvent compilations. Studies focussing solely on the design of ionic liquids or on the selection from a series of ionic liquids are not included.

Individual chemical species are frequently known by several synonyms. These include common or historical names and systematic or rational names, along with a small number of trade names or company product names. The CAS RN^(a)^ provides a convenient access to such synonyms and abbreviations.

Compounds in Table S2 are organised into a series of main chemical/functional group classes similar to those of Table S1. They are then listed according to their molecular formula, BC_t_H_u_X_v_N_w_O_x_P_y_S_z_, where X = F, Cl, Br, I.

Nearly all the compounds included are molecular liquids with boiling points in the range 25-300 °C. Some compounds have melting points close to or slightly above room temperature. Reports of the boiling points of many well-known compounds give a wide range of values of varying accuracy and reliability. The individual values reproduced in Table S1 and S2 are those reported in the headline CAS RN entry for the relevant compound. The CAS entry provides access to the source of this value and additional values. Where no published boiling points are available, CAS RN provides an estimated value.

The materials come from two types of basic literature sources: (i) lists of candidate compounds brought together as the bases for purposeful searches for solvents which might fulfil a particular function and (ii) sets of a range of chemicals meeting various physical characteristics to define a chemical ‘space’ from which one or more materials may be designed which optimise a set of more narrowly-defined criteria. While some compounds appear in more than one published source, one reference only is given for each entry. This has been selected arbitrarily.

The range of materials listed reflects the wide diversity of solvent application. Where studies to discover alternative solvents for a particular application have been fully described in the open literature it is clear that the exercise involves a broad initial search of potential candidates followed a gated approach which applies additional or more stringent criteria (such as toxicological, environmental and technocommercial) before identifying the preferred product. The initial set can include as many as 3000 potential candidates.^(b)^

The Solvent Selection Guides, Table S1, list 154 compounds in industrial use. By definition, these have been known as chemical species for some time. It might be thought, therefore, that those contained in Table S2 would include a significant number of new compounds. Perhaps surprisingly, reference to the final column of Table S2 shows that this is not the case. The final column lists the year in which the Chemical Abstract Service abstracts its first reference to the relevant named material. The great majority of compounds used in searches for alternative solvents have been known for 50 to >100 years. In addition to ‘neoteric’ solvents (ionic liquids, fluorous fluids and supercritical fluids), only about a dozen of what might be considered conventional alternatives have been added to CAS RN since 1980. Most of the latter arise from studies of biomass-derived precursors.

^(a)^ The Chemical Abstracts Service Registry Number (CAS RN) is a unique identifier for chemical substances the characteristics of which have been published and abstracted by the Chemical Abstracts Service of the American Chemical Society. The use of this number as a search term in the SciFinder® data base (<http://www.scifinder-n.cas.org>) provides links to associated chemical literature, key physical and chemical properties and other useful information. The unique identifier is particularly useful when an individual compound has a number of common, technical and scientific names.

^(b)^ Tickner et al (Tickner JA, Simon RV, Jacobs M, Pollard LD and van Bergen SK (2021) The nexus between alternatives assessment and green chemistry: supporting the development and adoption of safer chemicals. Green Chem Lett Rev 14:21-42) describe the development by Eastman Chemicals Co. of butyl 3-hydroxybutyrate (CAS RN = 53605-94-0, Table S2 entry 90) as the industrial cleaner, Omnia^TM^, which began with a database of ca. 3 000 possible candidates

|  | | **TABLE S2 ADDITIONAL SOLVENTS LISTED IN VARIOUS COMPILATIONS and PAPERS** | | | | | |  |
| --- | --- | --- | --- | --- | --- | --- | --- | --- |
|  |  | |  |  |  |  |  | |
| Entry No. | **SOLVENT** | | CAS Registry Number | Molecular Formula | Boiling Point (°C) | Ref. | Year of first  CAS report | |
|  |  | |  |  |  |  |  | |
|  | **ACIDS/ANHYDRIDES** | |  |  |  |  |  | |
| 1 | pyruvic acid | | 127-17-3 | C3H4O3 | 165 (mp 11-12) | Di Girolamo et al (2021) | 1878 | |
| 2 | 3-Hydroxypropionic acid | | 503-66-2 | C3H6O3 | 179-180.5 | Moity, Durand et al 2014 | 1893 | |
| 3 | methoxyacetic acid | | 625-45-6 | C3H6O3 | 203.5 | Di Girolamo et al (2021) | 1913 | |
| 4 | butyric acid | | 107-92-6 | C4H8O2 | 165.5 | Tobiszewski et al 2015 | 1879 | |
| 5 | isobutyric acid | | 79-31-2 | C4H8O2 | 152-155 | Tobiszewski et al 2015 | 1878 | |
| 6 | valeric acid | | 109-52-4 | C5H19O2 | 186-187 | Tobiszewski et al 2015 | 1879 | |
| 7 | hexanoic acid | | 142-62-1 | C6H12O2 | 205.8 | Tobiszewski et al 2015 | 1879 | |
| 8 | octanoic acid | | 124-07-2 | C8H16O2 | 239.7 | Wypych and Wypych 2014, 2019 | 1879 | |
| 9 | linoleic acid | | 60-33-3 | C18H32O2 | 365.2 | Wypych and Wypych 2014, 2019 | 1895 | |
| 10 | oleic acid | | 112-80-1 | C18H34O2 | 286/100 torr | Moity, Durand et al 2014 | 1878 | |
| 11 | ricinoleic acid | | 141-22-0 | C18H34O3 | 245/10 torr | Moity, Durand et al 2014 | 1907 | |
|  |  | |  |  |  |  |  | |
|  | **ALCOHOLS/DIOLS/TRIOL** | |  |  |  |  |  | |
| 12 | allyl alcohol | | 107-18-6 | C3H6O | 97 | Driver and Hunter 2020 | 1878 | |
| 13 | 3-pentanol | | 584-02-1 | C5H12O | 116 | Murray et al 2016 | 1914 | |
| 14 | 2-methyl-1-butanol | | 137-32-6 | C5H12O | 128 | Murray et al 2016 | 1909 | |
| 15 | 2-methyl-2-butanol | | 75-85-4 | C5H12O | 102.4 | Murray et al 2016 | 1884 | |
| 16 | 2-ethyl-1-butanol | | 97-95-0 | C6H14O | 146.3 | Murray et al 2016 | 1890 | |
| 17 | 2-methyl-1-pentanol | | 105-30-6 | C6H14O | 148 | Murray et al 2016 | 1911 | |
| 18 | 3-methyl-3-pentanol | | 77-74-7 | C6H14O | 122.4 | Murray et al 2016 | 1912 | |
| 19 | 4-methyl-2-pentanol | | 108-11-2 | C6H14O | 133 | Murray et al 2016 | 1909 | |
| 20 | 1-nonanol | | 143-08-8 | C9H20O | 213.4 | Diorazio et al 2016 | 1907 | |
| 21 | 1-decanol | | 112-30-1 | C10H22O | 232.9 | Moity, Durand et al 2014 | 1883 | |
| 22 | 1-dodecanol | | 112-53-8 | C12H26O | 259 | Driver and Hunter 2020 | 1893 | |
|  |  | |  |  |  |  |  | |
| 23 | 1,4-cineol | | 470-67-7 | C10H18O | 173-174 | Moity, Durand et al 2014 | 1945 | |
| 24 | 1,8-cineol | | 470-82-6 | C10H18O | 176.4 | Jessop et al 2012 | 1880 | |
| 25 | geraniol | | 106-24-1 | C10H18O | 230 | Moity, Durand et al 2014 | 1890 | |
| 26 | α-terpineol | | 98-55-5 | C10H18O | 218-221 | Moity, Durand et al 2014 | 1893 | |
| 27 | β-terpineol | | 138-87-4 | C10H18O | 210 | Moity, Durand et al 2014 | 1907 | |
| 28 | (±)-linalool | | 78-70-6 | C10H18O | 195-199 | Wypych and Wypych 2014, 2019 | 1898 | |
| 29 | α,3,3-trimethylcyclohexanemethanol | | 25225-09-6 | C10H20O | 100/10 torr | Moity, Durand et al 2014 | 1968 | |
| 30 | dihydromyrcenol | | 18479-58-8 | C10H20O | 92/20 torr | Moity, Durand et al 2014 | 1925 | |
| 31 | nopol | | 128-50-7 | C11H18O | 235 | Moity, Durand et al 2014 | 1944 | |
| 32 | oleyl alcohol | | 143-28-2 | C18H36O | 333 | Moity, Durand et al 2014 | 1924 | |
|  |  | |  |  |  |  |  | |
| 33 | 3-methoxy-3-methylbutan-1-ol | | 56539-66-3 | C6H14O2 | 175 | Moity, Durand et al 2014 | 1947 | |
| 34 | 2-methyl-2-(2-methylpropyl)-1,3-dioxolane-4-methanol | | 5660-53-7 | C8H18O3 | 72/0.1 torr | Moity, Durand et al 2014 | 1942 | |
|  |  | |  |  |  |  |  | |
| 35 | phenol | | 108-95-2 | C6H6O | 181.8 | Diorazio et al 2016 | 1850 | |
| 36 | o-cresol | | 95-48-7 | C7H8O | 191-192 | Driver and Hunter 2020 | 1884 | |
| 37 | m-cresol | | 108-39-4 | C7H8O | 202 | Driver and Hunter 2020 | 1869 | |
| 38 | p-cresol | | 106-44-5 | C7H8O | 201.8 (mp 35.5) | Driver and Hunter 2020 | 1864 | |
| 39 | 2,4-dimethylphenol | | 105-67-9 | C8H10O | 211.5 | Driver and Hunter 2020 | 1886 | |
| 40 | 2-phenylethanol | | 60-12-8 | C8H10O | 218.2 | Driver and Hunter 2020 | 1906 | |
|  |  | |  |  |  |  |  | |
| 41 | 1,2-butanediol | | 584-03-2 | C4H10O2 | 195-197 | Driver and Hunter 2020 | 1913 | |
| 42 | (2R,3S)-2,3-butanediol | | 5341-95-7 | C4H10O2 | 183.5 | Driver and Hunter 2020 | 1950 | |
|  |  | |  |  |  |  |  | |
| 43 | 2-methylenebutane-1,3-diol | | 55881-94-2 | C5H10O2 | 62-63/0.01 torr | Moity, Molinier et al 2014 | 1949 | |
| 44 | 1,2-pentanediol | | 5343-92-0 | C5H10O2 | 210-211 | Clark et al 2017a | 1923 | |
| 45 | 1,5-pentanediol | | 111-29-5 | C5H10O2 | 239 | Driver and Hunter 2020 | 1887 | |
|  |  | |  |  |  |  |  | |
|  | **KETONES/ALDEHYDES** | |  |  |  |  |  | |
| 46 | propionaldehyde | | 123-38-6 | C3H6O | 49 | Driver and Hunter 2020 | 1884 | |
| 47 | butyraldehyde | | 123-72-8 | C4H8O | 74.8 | Driver and Hunter 2020 | 1894 | |
| 48 | benzaldehyde | | 100-52-7 | C7H6O | 179 | Driver and Hunter 2020 | 1853 | |
| 49 | cinnamaldehyde | | 104-55-2 | C9H8O | 246 | Driver and Hunter 2020 | 1885 | |
|  |  | |  |  |  |  |  | |
| 50 | *iso*-propyl methyl ketone | | 563-80-4 | C5H10O | 93 | Diorazio et al 2016 | 1887 | |
| 51 | 3,3-dimethyl-2-butanone | | 75-97-8 | C6H12O | 106.1 | Pellis et al 2019 | 1879 | |
| 52 | 2-methylcyclohexanone | | 583-60-8 | C7H12O | 165.1 | Diorazio et al 2016 | 1908 | |
| 53 | ethyl butyl ketone | | 106-35-4 | C7H14O | 147 | Murray et al 2016 | 1927 | |
| 54 | methyl isoamyl ketone | | 110-12-3 | C7H14O | 144 | Murray et al 2016 | 1881 | |
| 55 | di-*iso*-propyl ketone | | 565-80-0 | C7H14O | 125.4 | Diorazio et al 2016 | 1887 | |
| 56 | 2-heptanone | | 110-43-0 | C7H14O | 151 | Driver and Hunter 2020 | 1908 | |
| 57 | acetophenone | | 98-86-2 | C8H8O | 202 | Murray et al 2016 | 1882 | |
| 58 | ethyl amyl ketone | | 106-68-3 | C8H16O | 167.5 | Murray et al 2016 | 1910 | |
| 59 | ethyl phenyl ketone | | 93-55-0 | C9H10O | 218 | Driver and Hunter 2020 | 1880 | |
| 60 | benzyl methyl ketone | | 103-79-7 | C9H10O | 216.5 | Driver and Hunter 2020 | 1902 | |
| 61 | isophorone | | 78-59-1 | C9H14O | 215.3 | Diorazio et al 2016 | 1902 | |
| 62 | di-*tert*-butyl ketone | | 815-24-7 | C9H18O | 152 | Murray et al 2016 | 1882 | |
| 63 | di-*n*-butyl ketone | | 502-56-7 | C9H18O | 188.4 | Diorazio et al 2016 | 1912 | |
| 64 | di-*sec*-butyl ketone | | 19549-84-9 | C9H18O | 162 | Diorazio et al 2016 | 1923 | |
| 65 | di-*iso*-butyl ketone | | 108-83-8 | C9H18O | 168 | Diorazio et al 2016 | 1909 | |
|  |  | |  |  |  |  |  | |
| 66 | 2,3-butanedione | | 431-03-8 | C4H6O2 | 88 | Driver and Hunter 2020 | 1885 | |
| 67 | acetylacetone | | 123-54-6 | C5H8O2 | 140 | Diorazio et al 2016 | 1881 | |
| 68 | diacetone alcohol | | 123-42-2 | C6H12O2 | 170 | Diorazio et al 2016 | 1900 | |
| 69 | 5-(hydroxymethyl)furfural | | 67-47-0 | C6H6O3 | 115/0.5 torr (mp 31.5) | Moity, Durand et al 2014 | 1910 | |
| 70 | 1-(2-furanyl)-4-methyl-1-penten-3-one | | 4996-48-9 | C10H14O3 | 250.2±13.0 (est) | www.xftechnologies.com/solvents | 1965 | |
|  |  | |  |  |  |  |  | |
|  | **ESTERS/ORTHOESTERS** | |  |  |  |  |  | |
| 71 | propyl formate | | 110-74-7 | C4H8O2 | 80.9 | Diorazio et al 2016 | 1884 | |
| 72 | ethyl acrylate | | 140-88-5 | C5H8O2 | 99.4 | Tobiszewski et al 2015 | 1891 | |
| 73 | butyl formate | | 592-84-7 | C5H10O2 | 107 | Diorazio et al 2016 | 1880 | |
| 74 | methyl isobutyrate | | 547-63-7 | C5H10O2 | 92.5 | Murray et al 2016 | 1893 | |
| 75 | methyl butyrate | | 623-42-7 | C5H10O2 | 102.8 | Diorazio et al 2016 | 1884 | |
| 76 | 2-butyl acetate | | 105-46-4 | C6H12O2 | 112 | Murray et al 2016 | 1893 | |
| 77 | methyl pivalate | | 598-98-1 | C6H12O2 | 101.1 | Byrne et al 2018 | 1911 | |
| 78 | ethyl butanoate | | 105-54-4 | C6H12O2 | 120-121 | Murray et al 2016 | 1879 | |
| 79 | hexyl acetate | | 142-92-2 | C8H16O2 | 171.5 | Diorazio et al 2016 | 1894 | |
| 80 | butyl butyrate | | 109-21-7 | C8H16O2 | 166 | Murray et al 2016 | 1915 | |
| 81 | methyl caprylate | | 111-11-5 | C9H18O2 | 192.9 | Tobiszewski et al 2015 | 1914 | |
|  |  | |  |  |  |  |  | |
| 82 | methyl lactate | | 547-64-8 | C4H8O3 | 144-145 | Di Girolamo et al (2021) | 1895 | |
| 83 | methyl acetoacetate | | 105-45-3 | C5H8O3 | 172 (mp 27-28) | Di Girolamo et al (2021) | 1883 | |
| 84 | ethyl acetoacetate | | 141-97-9 | C6H10O3 | 180.8 | Diorazio et al 2016 | 1878 | |
| 85 | *iso*-propyl lactate | | 617-51-6 | C6H12O3 | 167 | Wypych and Wypych 2014, 2019 | 1930 | |
| 86 | methyl levulinate | | 624-45-3 | C6H10O3 | 192-193 | Marcel et al 2019 | 1891 | |
| 87 | 2-ethoxyethyl acetate | | 111-15-9 | C6H12O3 | 156 | Diorazio et al 2016 | 1914 | |
| 88 | ethyl levulinate | | 539-88-8 | C7H12O3 | 205.8 | Ho et al 2020 | 1898 | |
| 89 | butyl lactate | | 138-22-7 | C7H14O3 | 186 | Tobiszewski et al 2015 | 1926 | |
| 90 | butyl 3-hydroxybutyrate | | 53605-94-0 | C8H16O3 | 241.7±13.0 (est) | Clark et al 2017a | 1974 | |
| 91 | butyl levulinate | | 2052-15-5 | C9H16O3 | 237.5 | Marcel et al 2019 | 1930 | |
| 92 | *sec*-butyl-4-oxopentanoate | | 85734-01-6 | C9H16O3 | 225.8 | Ho et al 2020 | 1933 | |
| 93 | pentyl-4-oxopentanoate | | 20729-49-6 | C10H18O3 | 259.0±13.0 (est) | Ho et al 2020 | 1931 | |
| 94 | ethylhexyl lactate | | 6283-86-9 | C11H22O3 | 97-98/3 torr | Moity, Durand et al 2014 | 1949 | |
| 95 | methyl ricinoleate | | 141-24-2 | C19H36O3 | 245/10 torr | Moity, Durand et al 2014 | 1907 | |
|  |  | |  |  |  |  |  | |
| 96 | methyl 5-methyl-2-furancarboxylate | | 2527-96-0 | C7H8O3 | 205 | www.xftechnologies.com/solvents | 1930 | |
| 97 | ethyl 5-methyl-2-furancarboxylate | | 14003-12-4 | C8H10O3 | 71/4 torr | www.xftechnologies.com/solvents | 1934 | |
| 98 | *iso*-propyl 5-methyl-2-furancarboxylate | | 7042-05-9 | C9H12O3 | 130-131/14 torr | www.xftechnologies.com/solvents | 1966 | |
| 99 | pentyl 2-furancarboxylate | | 4996-48-9 | C10H14O3 | 250.2±13.0 (est) | Bergez-Lacoste et al 2014 | 1965 | |
| 100 | 2-ethylhexyl 2-methylfuranoate | | 1687726-83-5 | C14H22O3 | 318.2±22.0 (est) | www.xftechnologies.com/solvents | - | |
| 101 | 2-hydroxymethyl 2-methylfuranoate | | 1864952-99-7 | C8H10O4 | 295.9±30.0 (est) | www.xftechnologies.com/solvents | - | |
| 102 | 2-methoxyethyl 2-methylfuranoate | | 1850242-83-9 | C9H12O4 | 235.9±25.0 (est) | www.xftechnologies.com/solvents | - | |
|  |  | |  |  |  |  |  | |
| 103 | dimethyl malonate | | 108-59-8 | C5H8O4 | 181.4 | Di Girolamo et al (2021) | 1881 | |
| 104 | diethyl malonate | | 105-53-3 | C7H12O4 | 200 | Driver and Hunter 2020 | 1882 | |
| 105 | dimethyl glutarate | | 1119-40-0 | C7H12O4 | 93.5-94.5/13 torr | Clark et al 2017a | 1891 | |
| 106 | 1,5-dimethyl 2-methylpentanedioate | | 14035-94-0 | C8H14O4 | 104/14 torr | Wypych and Wypych 2014, 2019 | 1929 | |
| 107 | diethyl glutarate | | 818-38-2 | C9H16O4 | 236.5 | Moity, Durand et al 2014 | 1887 | |
| 108 | diethyl adipate | | 141-28-6 | C10H18O4 | 245 | Moity, Durand et al 2014 | 1887 | |
| 109 | di-*iso*-butyl succinate | | 925-06-4 | C12H22O4 | 216 | Moity, Durand et al 2014 | 1884 | |
| 110 | di-*iso*-butyl glutarate | | 71195-64-7 | C13H24O4 | 237 | Moity, Durand et al 2014 | 1982 | |
| 111 | di-*iso*-amyl succinate | | 818-04-2 | C14H26O4 | 281.1±8.0 (est) | Moity, Durand et al 2014 | 1979 | |
| 112 | di-*iso*-butyl adipate | | 141-04-8 | C14H26O4 | 278-280 | Moity, Durand et al 2014 | 1937 | |
| 113 | di-butyl sebacate | | 109-43-3 | C18H34O4 | 344.5 | Moity, Durand et al 2014 | 1945 | |
| 114 | dioctyl succinate | | 14491-66-8 | C20H38O4 | 210/0.11 torr | Moity, Durand et al 2014 | 1954 | |
| 115 | di-*iso*-octyl succinate | | 2915-57-3 | C20H38O4 | 206-208/4 torr | Moity, Durand et al 2014 | 1936 | |
|  |  | |  |  |  |  |  | |
| 116 | methyl benzoate | | 93-58-3 | C8H8O2 | 198-200 | Driver and Hunter 2020 | 1881 | |
| 117 | ethyl benzoate | | 93-89-0 | C9H10O2 | 212 | Driver and Hunter 2020 | 1880 | |
| 118 | benzyl benzoate | | 120-51-4 | C14H12O2 | 323-324 | Moity, Durand et al 2014 | 1899 | |
| 119 | dimethyl phthalate | | 131-11-3 | C10H10O4 | 283.7 | Driver and Hunter 2020 | 1886 | |
| 120 | diethyl phthalate | | 84-66-2 | C12H14O4 | 298 | Moity, Durand et al 2014 | 1886 | |
|  |  | |  |  |  |  |  | |
| 121 | neryl acetate | | 141-12-8 | C12H20O2 | 235 | Tobiszewski et al 2015 | 1929 | |
| 122 | geranyl acetate | | 105-87-3 | C12H20O2 | 240 | Moity, Durand et al 2014 | 1929 | |
| 123 | terpineol acetate | | 8007-35-0 | C12H20O2 | 104-106/11 torr | Moity, Durand et al 2014 | 1902 | |
| 124 | menthanyl acetate | | 58985-18-5 | C12H22O2 |  | Moity, Durand et al 2014 | 1975 | |
| 125 | methyl laurate | | 111-82-0 | C13H26O2 | 267 | Ho et al 2020 | 1914 | |
| 126 | ethyl laurate | | 106-33-2 | C14H28O2 | 271 | Moity, Durand et al 2014 | 1928 | |
| 127 | methyl myristate | | 124-10-7 | C15H30O2 | 295 | Moity, Durand et al 2014 | 1914 | |
| 128 | ethyl myristate | | 124-06-1 | C16H32O2 | 295 | Moity, Durand et al 2014 | 1930 | |
| 129 | methyl palmitate | | 112-39-0 | C17H34O2 | 417 | Moity, Durand et al 2014 | 1913 | |
| 130 | *iso*-propyl myristate | | 110-27-0 | C17H34O2 | 192.6/20 torr | Moity, Durand et al 2014 | 1942 | |
| 131 | ethyl palmitate | | 628-97-7 | C18H36O2 | 189-192/10 torr | Moity, Durand et al 2014 | 1913 | |
| 132 | butyl myristate | | 110-36-1 | C18H36O2 | 271-273 | Moity, Durand et al 2014 | 1927 | |
| 133 | methyl stearate | | 112-61-8 | C19H38O2 | 443 | Moity, Durand et al 2014 | 1907 | |
| 134 | methyl linolenate | | 301-00-8 | C19H32O2 | 182 | Moity, Durand et al 2014 | 1935 | |
| 135 | methyl linoleate | | 112-63-0 | C19H34O2 | 215 | Moity, Durand et al 2014 | 1923 | |
| 136 | *iso*-propyl palmitate | | 142-91-6 | C19H38O2 | 160/2 torr | Moity, Durand et al 2014 | 1942 | |
| 137 | ethyl linoleate | | 544-35-4 | C20H36O2 | 205-206/10 torr | Moity, Durand et al 2014 | 1927 | |
| 138 | ethyl linolenate | | 1191-41-9 | C20H34O2 | 197/6.5 torr | Moity, Durand et al 2014 | 1929 | |
| 139 | ethyl oleate | | 111-62-6 | C20H38O2 | 195-200/10 torr | Moity, Durand et al 2014 | 1884 | |
| 140 | butyl palmitate | | 111-06-8 | C20H40O2 | 362.4±10.0 (est) | Moity, Durand et al 2014 | 1927 | |
| 141 | methyl abietate | | 127-25-3 | C21H32O2 | 362.5 | Moity, Durand et al 2014 | 1934 | |
| 142 | methyl arachidonate | | 2566-89-4 | C21H34O2 | 200-205 /1-2 torr | Wypych and Wypych 2014, 2019 | 1934 | |
| 143 | butyl stearate | | 123-95-5 | C22H44O2 | 343 | Moity, Durand et al 2014 | 1927 | |
| 144 | octyl palmitate | | 29806-73-3 | C24H48O2 | 407.2±13.0 (est)/(mp 2) | Wypych and Wypych 2014, 2019 | 1970 | |
|  |  | |  |  |  |  |  | |
| 145 | methyl orthoacetate | | 1445-45-0 | C5H12O3 | 108 | Driver and Hunter 2020 | 1944 | |
| 146 | triethyl orthoformate | | 122-51-0 | C7H16O3 | 143 | Diorazio et al 2016 | 1884 | |
| 147 | tetraethyl orthocarbonate | | 1850-14-2 | C5H12O4 | 114 | Diorazio et al 2016 | 1927 | |
| 148 | methyl tetrafurfuryl carbonate | | 288148-60-7 | C7H12O4 | 211.6±13.0 (est) | Ho et al 2020 | 2000 | |
| 149 | ethylene glycol monomethyl ether acetate | | 110-49-6 | C5H10O3 | 145 | Wypych and Wypych 2014, 2019 | 1910 | |
| 150 | 1-methoxy-2-propyl acetate | | 108-65-6 | C6H12O3 | 146 | Wypych and Wypych 2014, 2019 | 1930 | |
| 151 | diethylene glycol monomethyl ether acetate | | 629-38-9 | C7H14O4 | 85-95 /0.2 torr | Wypych and Wypych 2014, 2019 | 1948 | |
| 152 | glycol monobutyl ether acetate | | 112-07-2 | C8H16O3 | 192.3 | Wypych and Wypych 2014, 2019 | 1933 | |
| 153 | 2-(2-ethoxyethoxy)ethyl acetate | | 112-15-2 | C8H16O4 | 218.5 | Wypych and Wypych 2014, 2019 | 1937 | |
| 154 | dipropylene glycol methyl ether acetate | | 88917-22-0 | C9H18O4 |  | Wypych and Wypych 2014, 2019 | 1984 | |
| 155 | diethylene glycol monobutyl ether acetate | | 124-17-4 | C10H20O4 | 245 | Wypych and Wypych 2014, 2019 | 1939 | |
|  |  | |  |  |  |  |  | |
| 156 | *iso*-sorbide dioctanoate | | 64896-70-4 | C22H38O6 | 476.7±45.0 (est) | Moity, Durand et al 2014 | 1953 | |
| 157 | glycerol trioleate | | 122-32-7 | C57H104O6 | 235-240/15 torr | Jessop et al 2012 | 1893 | |
| 158 | triethyl citrate | | 77-93-0 | C12H20O7 | 294 | Moity, Durand et al 2014 | 1887 | |
| 159 | tributyl citrate | | 77-94-1 | C18H32O7 | 170-180/11 torr | Moity, Durand et al 2014 | 1941 | |
| 160 | acetyltributyl citrate | | 77-90-7 | C20H34O8 | 172-174/1 torr | Moity, Durand et al 2014 | 1951 | |
|  |  | |  |  |  |  |  | |
|  | **ETHERS/ACETALS/KETALS/ETHER ALCOHOLS** | |  |  |  |  |  | |
| 161 | dimethyl ether | | 115-10-6 | C2H6O | -24.8 | Bauer and Kruse 2019 | 1851 | |
| 162 | furan | | 110-00-9 | C4H4O | 31.4 | Diorazio et al 2016 | 1886 | |
| 163 | tetrahydropyran | | 142-68-7 | C5H10O | 88 | Murray et al 2016 | 1913 | |
| 164 | *n*-butyl methyl ether | | 628-28-4 | C5H10O | 70.1 | Murray et al 2016 | 1894 | |
| 165 | 2,5-dimethylfuran | | 625-86-5 | C6H8O | 93.5 | Hu et al 2014 | 1910 | |
| 166 | 2,5-dimethyltetrahydrofuran | | 1003-38-9 | C6H12O | 90-92 | Yang and Sen 2010 | 1907 | |
| 167 | 4-methyltetrahydropyran | | 4717-96-8 | C6H12O | 105 | Kobayashi et al 2019 | 1960 | |
| 168 | di-*n*-propyl ether | | 111-43-3 | C6H14O | 90 | Murray et al 2016 | 1880 | |
| 169 | ethyl *n*-butyl ether | | 628-81-9 | C6H14O | 92.3 | Murray et al 2016 | 1894 | |
| 170 | 2-methylanisole | | 578-58-5 | C8H10O | 171 | Lee et al 2017 | 1884 | |
| 171 | 2-butyltetrahydrofuran | | 1004-29-1 | C8H16O | 159-160 | Strohmann et al 2019 | 1914 | |
| 172 | 2,2,5,5-tetramethyltetrahydrofuran | | 15045-43-9 | C8H16O | 112 | Byrne et al 2017 | 1915 | |
| 173 | (+)-rose oxide | | 16409-43-1 | C10H18O | 58/12 torr | Gevorgyan et al 2020 | 1961 | |
| 174 | dipentyl ether | | 693-65-2 | C10H22O | 190 | Murray et al 2016 | 1894 | |
| 175 | dibenzyl ether | | 103-50-4 | C14H14O | 298 | Driver and Hunter 2020 | 1878 | |
|  |  | |  |  |  |  |  | |
| 176 | 1,3-dioxan | | 505-22-6 | C4H8O2 | 105 | Driver and Hunter 2020 | 1912 | |
| 177 | dimethyl acetal | | 534-15-6 | C4H10O2 | 64.5 | Gevorgyan et al 2020 | 1884 | |
| 178 | 1-methoxy-2-propanol | | 107-98-2 | C4H10O2 | 120.1 | Diorazio et al 2016 | 1930 | |
| 179 | 2,2-dimethoxypropane | | 77-76-9 | C5H12O2 | 83 | Diorazio et al 2016 | 1934 | |
| 180 | 2,2-dimethyl-1,3-dioxolane | | 2916-31-6 | C5H10O2 | 92.5 | Diorazio et al 2016 | 1929 | |
| 181 | acetaldehyde diethyl acetal | | 105-57-7 | C6H14O2 | 103.7-104 | Murray et al 2016 | 1859 | |
| 182 | 2-methoxyphenol | | 90-05-1 | C7H8O2 | 204-206 (mp 32) | Driver and Hunter 2020 | 1998 | |
| 183 | 1,2-dimethoxybenzene | | 91-16-7 | C8H10O2 | 206 (mp 22.5) | Driver and Hunter 2020 | 1897 | |
| 184 | dibutoxymethane | | 2568-90-3 | C9H20O2 | 179.2 °C | Jessop et al 2012 | 1924 | |
|  |  | |  |  |  |  |  | |
| 185 | 1,1'-oxybis(1-methoxymethane) | | 628-90-0 | C4H10O3 | 121.5-122/13 torr | Zhenova et al 2019 | 1928 | |
| 186 | trimethyl orthoformate | | 149-73-5 | C4H10O3 | 100.6 | Diorazio et al 2016 | 1932 | |
| 187 | 3-methoxy-1,2-propanediol | | 623-39-2 | C4H10O3 | 220 | Moity, Durand et al 2014 | 1909 | |
| 188 | 2-methoxy-1,3-propanediol | | 761-06-8 | C4H10O3 | 122-123/14 torr | Moity, Durand et al 2014 | 1928 | |
|  |  | |  |  |  |  |  | |
| 189 | glycerol-1,2-dimethyl ether | | 40453-77-8 | C5H12O3 | 69.5-70.5/15 torr | Pellis et al 2019 | 1925 | |
| 190 | glycerol-1,3-dimethyl ether | | 623-69-8 | C5H12O3 | 169 | Strohmann et al 2019 | 1929 | |
| 191 | glycerol-1-ethyl monoether | | 1874-62-0 | C5H12O3 | 160/100 torr | Moity, Durand et al 2014 | 1926 | |
| 192 | glycerol-2-ethyl monoether | | 22598-16-9 | C5H12O3 | 90-92/12 torr | Moity, Durand et al 2014 | 1926 | |
| 193 | 2-(2-ethoxyethoxy)ethanol | | 111-90-0 | C6H14O3 | 196 | Shakeel et al 2014 | 1930 | |
| 194 | bis(2-hydroxypropyl) ether | | 110-98-5 | C6H14O3 | 233 | Moity, Durand et al 2014 | 1927 | |
| 195 | glycerol *iso*-butyral | | 31193-94-6 | C7H14O3 | 209.3±15.0 | Estévez 2009 | 1937 | |
| 196 | glycerol-1,2-diethyl ether | | 4756-20-1 | C7H16O3 | 220.2±20.0 | Moity, Durand et al 2014 | 1930 | |
| 197 | glycerol-1,3-diethyl ether | | 4043-59-8 | C7H16O3 | 61.5-63/2 torr | Moity, Durand et al 2014 | 1930 | |
| 198 | glycerol-1-butyl monoether | | 624-52-2 | C7H16O3 | 226-227 | Jessop et al 2012 | 1930 | |
| 199 | glycerol-2-butyl monoether | | 100078-36-2 | C7H16O3 | 260.3±20.0 (est) | Moity, Durand et al 2014 | 1981 | |
| 200 | 1-methoxy-3-*iso*-propoxy-2-propanol | | 53146--34-2 | C7H16O3 | 202-204 | Tobiszewski et al 2015 | 1957 | |
| 201 | diethylene glycol diether | | 112-36-7 | C8H18O3 | 188 | Jessop et al 2012 | 1925 | |
| 202 | 1-ethoxy-3-*iso*-propoxy-2-propanol | | 13021-50-6 | C8H18O3 | 207-209 | Tobiszewski et al 2015 | 1949 | |
| 203 | 1-*n*-butoxy-3-methoxy-2-propanol | | 13021-51-7 | C8H18O3 | 208 | Tobiszewski et al 2015 | 1962 | |
| 204 | 1-*tert*-butoxy-3-methoxy-2-propanol | | 13021-53-9 | C8H18O3 | 195 | Tobiszewski et al 2015 | 1962 | |
| 205 | 1-*n*-butoxy-3-ethoxy-2-propanol | | 13021-52-8 | C9H20O3 | 220 | Tobiszewski et al 2015 | 1954 | |
| 206 | 1-*tert*-butoxy-3-ethoxy-2-propanol | | 42910-64-5 | C9H20O3 | 204 | Tobiszewski et al 2015 | 1954 | |
| 207 | 1,3-di-*iso*-propoxy-2-propanol | | 13021-54-0 | C9H20O3 | 202 | Tobiszewski et al 2015 | 1930 | |
| 208 | 1,2,3-triethoxypropane | | 162614-45-1 | C9H20O3 | 78=80/15 torr | Qian et al 2021 | 1947 | |
| 209 | 1-*n*-butoxy-3-*iso*-propoxy-2-propanol | | 53146-43-3 | C10H22O3 | 223 | Tobiszewski et al 2015 | 1974 | |
| 210 | glycerol-1,2-dibutyl ether | | 91337-36-9 | C11H24O3 | 293.2±20.0 (est) | Moity, Durand et al 2014 | 1962 | |
| 211 | 3-*n*-butoxy-1-*tert*-butoxy-2-propanol | | 42910-65-6 | C11H24O3 | 230 | Tobiszewski et al 2015 | 1973 | |
| 212 | 1,3-di-*n*-butoxy-2-propanol | | 2216-77-5 | C11H24O3 | 248 | Tobiszewski et al 2015 | 1939 | |
| 213 | 1,2,3-tri-*n*-butoxypropane | | 131570-29-1 | C15H32O3 | 270 | Tobiszewski et al 2015 | 1947 | |
|  |  | |  |  |  |  |  | |
| 214 | glycerol carbonate | | 931-40-8 | C4H6O4 | 130/0.08 torr | Christy et al 2018 | 1934 | |
| 215 | tetraglycol | | 52814-38-7 | C9H18O4 | 130/0.6 torr | Moity, Durand et al 2014 | 1955 | |
| 216 | methyl (2,2-dimethyl-1,3-dioxolan-4- yl)methyl carbonate | | 1354053-49-8 | C8H14O5 | 223.9±15.0 (est) | Jin et al 2017 | 2012 | |
|  |  | |  |  |  |  |  | |
|  | **HYDROCARBONS** | |  |  |  |  |  | |
| 217 | 1-pentene | | 109-67-1 | C5H10 | 29.9 | Tobiszewski et al 2015 | 1908 | |
| 218 | *cyclo*-pentane | | 287-92-3 | C5H10 | 49.2 | Diorazio et al 2016 | 1999 | |
| 219 | *iso*-pentane | | 78-78-4 | C5H12 | 27.8 | Driver and Hunter 2020 | 1884 | |
| 220 | 1-hexene | | 592-41-6 | C6H12 | 63.4 | Tobiszewski et al 2015 | 1911 | |
| 221 | methylcyclopentane | | 96-37-7 | C6H12 | 71.8 | Diorazio et al 2016 | 1890 | |
| 222 | 1-heptene | | 592-76-7 | C7H14 | 93.6 | Tobiszewski et al 2015 | 1926 | |
| 223 | 1-octene | | 111-66-0 | C8H16 | 121.1 | Tobiszewski et al 2015 | 1893 | |
| 224 | 1-nonene | | 124-11-8 | C9H18 | 146.9 | Tobiszewski et al 2015 | 1915 | |
| 225 | nonane | | 111-84-2 | C9H20 | 150.8 | Tobiszewski et al 2015 | 1884 | |
| 226 | γ-terpinene | | 99-85-4 | C10H16 | 183 | Gevorgyan et al 2020 | 1909 | |
| 227 | terpinolene | | 586-62-9 | C10H16 | 185 | Moity, Durand et al 2014 | 1879 | |
| 228 | (±)-α-pinene | | 80-56-8 | C10H16 | 156-161 | Gevorgyan et al 2020 | 1854 | |
| 229 | α-pinene | | 80-56-8 | C10H16 | 156-161 | Moity, Durand et al 2014 | 1854 | |
| 230 | β-pinene | | 127-91-3 | C10H16 | 166 | Moity, Durand et al 2014 | 1882 | |
| 231 | pinane (7:3 *cis:trans*) | | 473-55-2 | C10H18 | 169 | Yara-Varon et al 2016 | 1908 | |
| 232 | decane | | 124-18-5 | C10H22 | 174.1 | Murray et al 2016 | 1884 | |
| 233 | β-myrcene | | 123-35-3 | C10H36 | 167 | Moity, Durand et al 2014 | 1903 | |
| 234 | 3-carene | | 13466-78-9 | C10H16 | 170 | Tobiszewski et al 2015 | 1920 | |
| 235 | undecane | | 1120-21-4 | C11H24 | 195.9 | Tobiszewski et al 2015 | 1912 | |
| 236 | dodecane | | 112-40-3 | C12H26 | 216.3 | Driver and Hunter 2020 | 1897 | |
| 237 | *iso*-dodecane | | 31807-55-3 | C12H26 | 205-230 | Moity, Durand et al 2014 | 1942 | |
| 238 | tridecane | | 629-50-5 | C13H28 | 235.4 | Tobiszewski et al 2015 | 1924 | |
| 239 | tetradecane | | 629-59-4 | C14H30 | 253.7 | Tobiszewski et al 2015 | 1927 | |
| 240 | β-farnesene | | 18794-84-8 | C15H24 | 80-82/0.5 torr | Moity, Durand et al 2014 | 1963 | |
| 241 | farnesane (hydrogenated β-farnesene) | | 3891-98-3 | C15H32 | 119.5-120/11 torr | Clark et al 2017a | 1928 | |
| 242 | pentadecane | | 629-62-9 | C15H32 | 270.6 | Tobiszewski et al 2015 | 1924 | |
| 243 | hexadecane | | 544-76-3 | C16H34 | 286.5 | Driver and Hunter 2020 | 1885 | |
|  |  | |  |  |  |  |  | |
| 244 | styrene | | 100-42-5 | C8H8 | 145 | Driver and Hunter 2020 | 1879 | |
| 245 | o-xylene | | 95-47-6 | C8H10 | 144.4 | Driver and Hunter 2020 | 1879 | |
| 246 | m-xylene | | 108-38-3 | C8H10 | 139.3 | Driver and Hunter 2020 | 1879 | |
| 247 | ethylbenzene | | 100-41-4 | C8H14 | 136.1 | Diorazio et al 2016 | 1879 | |
| 248 | indane | | 496-11-7 | C9H10 | 177.9 | Diorazio et al 2016 | 1884 | |
| 249 | butyl benzene | | 104-51-8 | C10H14 | 183.3 | Diorazio et al 2016 | 1892 | |
|  |  | |  |  |  |  |  | |
|  | **WITH HETEROATOMS OTHER THAN OXYGEN** | |  |  |  |  |  | |
| 250 | butanenitrile | | 109-74-0 | C4H7N | 117.5 | Murray et al 2016 | 1879 | |
| 251 | pyrrolidine | | 123-75-1 | C4H9N | 86.7 | Murray et al 2016 | 1894 | |
| 252 | butylamine | | 109-73-9 | C4H11N | 78 | Di Girolamo et al (2021) | 1872 | |
| 253 | diethylamine | | 109-89-7 | C4H11N | 55.5 | Murray et al 2016 | 1878 | |
| 254 | *tert*-butylamine | | 75-64-9 | C4H11N | 44 | Diorazio et al 2016 | 1896 | |
| 255 | *tert-*butyl isocyanide | | 7188-38-7 | C5H9N | 91 | Di Girolamo et al (2021) | 1907 | |
| 256 | piperidine | | 110-89-4 | C5H11N | 106 | Diorazio et al 2016 | 1881 | |
| 257 | hexanenitrile | | 628-73-9 | C6H11N | 163.6 | Diorazio et al 2016 | 1884 | |
| 258 | *cyclo*-hexylamine | | 108-91-8 | C6H13N | 134.5 | Diorazio et al 2016 | 1903 | |
| 259 | benzonitrile | | 100-47-0 | C7H5N | 190.7 | Murray et al 2016 | 1879 | |
| 260 | phenylacetonitrile | | 140-29-4 | C8H7N | 233.5 | Driver and Hunter 2020 | 1880 | |
| 261 | tripropylamine | | 102-69-2 | C9H21N | 156 | Murray et al 2016 | 1889 | |
| 262 | tributylamine | | 102-82-9 | C12H27N | 216.5 | Diorazio et al 2016 | 1919 | |
|  |  | |  |  |  |  |  | |
| 263 | aniline | | 62-53-3 | C6H7N | 184.1 | Diorazio et al 2016 | 1866 | |
| 264 | 2-methylpyridine | | 109-06-8 | C6H7N | 128-129 | Diorazio et al 2016 | 1885 | |
| 265 | 3-methylpyridine | | 108-99-6 | C6H7N | 143-144 | Driver and Hunter 2020 | 1885 | |
| 266 | 4-methylpyridine | | 108-89-4 | C6H7N | 145 | Driver and Hunter 2020 | 1907 | |
| 267 | *N*-methyl aniline | | 100-61-8 | C7H9N | 196 | Diorazio et al 2016 | 1877 | |
| 268 | benzylamine | | 100-46-9 | C7H9N | 185 | Diorazio et al 2016 | 1882 | |
| 269 | 2,4-dimethylpyridine | | 108-47-4 | C7H9N | 158.5 | Driver and Hunter 2020 | 1902 | |
| 270 | 2,6-dimethylpyridine | | 108-45-5 | C7H9N | 144 | Murray et al 2016 | 1891 | |
| 271 | *N-*ethylaniline | | 103-69-5 | C8H11N | 204.5 | Diorazio et al 2016 | 1880 | |
| 272 | quinoline | | 91-22-5 | C9H7N | 237.7 | Murray et al 2016 | 1882 | |
| 273 | 4-*tert*-butylpyridine | | 3978-81-2 | C9H13N | 196.5 | Di Girolamo et al (2021) | 1951 | |
| 274 | *N*-methyl-*N*-phenylaniline | | 552-82-9 | C13H13N | 293.5 | Driver and Hunter 2020 | 1879 | |
|  |  | |  |  |  |  |  | |
| 275 | 2-chloropyridine | | 109-09-1 | C5H4ClN | 170 | Driver and Hunter 2020 | 1898 | |
| 276 | 2-bromopyridine | | 109-04-6 | C5H4BrN | 194.8 | Driver and Hunter 2020 | 1932 | |
|  |  | |  |  |  |  |  | |
| 277 | ethylenediamine | | 107-15-3 | C2H8N2 | 116-117 | Diorazio et al 2016 | 1884 | |
| 278 | dimethylcyanamide | | 1467-79-4 | C3H6N2 | 163.5 | Driver and Hunter 2020 | 1912 | |
| 279 | 1.3-diaminopropane | | 109-76-2 | C3H10N2 | 140 | Di Girolamo et al (2021) | 1909 | |
| 280 | 1-methylimidazole | | 616-47-7 | C4H6N2 | 195.5 | Murray et al 2016 | 1912 | |
| 281 | glutaronitrile | | 544-13-8 | C5H6N2 | 286 | Di Girolamo et al (2021) | 1889 | |
| 282 | 2-methylpyrazine | | 109-08-0 | C5H6N2 | 137 | Di Girolamo et al (2021) | 1950 | |
| 283 | 2-cyanopyridine | | 100-70-9 | C6H4N2 | 224.5 | Driver and Hunter 2020 | 1932 | |
| 284 | adiponitrile | | 111-69-3 | C6H8N2 | 295 | Diorazio et al 2016 | 1909 | |
| 285 | formamide | | 75-12-7 | CH3NO | 210.5 | Murray et al 2016 | 1882 | |
| 286 | acetaldoxime | | 107-29-9 | C2H5NO | 114 [mp 45 (form a); 12(form b)] | Di Girolamo et al (2021) | 1887 | |
| 287 | 2-aminoethanol | | 141-43-5 | C2H7NO | 170.8 | Murray et al 2016 | 1912 | |
| 288 | isoxazole | | 288-14-2 | C3H3NO | 95 | Di Girolamo et al (2021) | 1900 | |
| 289 | 2-cyanoethanol | | 109-78-4 | C3H5NO | 221 | Driver and Hunter 2020 | 1917 | |
| 290 | *N*-methylacetamide | | 79-16-3 | C3H7NO | 205 | Diorazio et al 2016 | 1910 | |
| 291 | 2-methoxyethylamine | | 109-85-3 | C3H9NO | 118-120 | Di Girolamo et al (2021) | 1920 | |
| 292 | 3-methoxypropionitrile | | 110-67-8 | C4H7NO | 163 | Di Girolamo et al (2021) | 1923 | |
| 293 | 2-pyrrolidone | | 616-45-5 | C4H7NO | 76/0.2 torr | Moity, Durand et al 2014 | 1890 | |
| 294 | morpholine | | 110-91-8 | C4H9NO | 128.9 | Diorazio et al 2016 | 1912 | |
| 295 | *N.N*-diethylhydroxylamine | | 3710-84-7 | C4H11NO | 133 (mp 10) | Di Girolamo et al (2021) | 1901 | |
| 296 | 4-methylmorpholine | | 109-02-4 | C5H11NO | 116 | Diorazio et al 2016 | 1938 | |
| 297 | diethylformamide | | 617-84-5 | C5H11NO | 177.5 | Driver and Hunter 2020 | 1927 | |
| 298 | *N*-methylcaprolactam | | 2556-73-2 | C7H13NO | 104-106/11 torr | Diorazio et al 2016 | 1921 | |
| 299 | *N*-butylpyrrolidinone | | 3470-98-2 | C8H15NO | 121/16 torr | Sherwood et al 2016 | 1942 | |
| 300 | *N*-heptyl-5-methyl-2-pyrrolidone | | 69343-70-0 | C11H21NO | 94-95/1.0 torr | Barbaro et al 2020 | 1954 | |
| 301 | *N,N*-dimethyl-9-decenamide | | 1356964-77-6 | C12H23NO | 291.8±19.0 (est) | Clark et al 2017a | 2010 | |
| 302 | *N,N*-dimethyldodecanamide | | 3007-53-2 | C14H29NO | 284.9±8.0 (est) | Wypych and Wypych 2014, 2019 | 1962 | |
| 303 | nitroethane | | 79-24-3 | C2H5NO2 | 114-115 | Driver and Hunter 2020 | 1884 | |
| 304 | 1-nitropropane | | 108-03-2 | C3H7NO2 | 131.6 | Driver and Hunter 2020 | 1933 | |
| 305 | 2-nitropropane | | 79-46-9 | C3H7NO2 | 120.3 | Diorazio et al 2016 | 1915 | |
| 306 | methyl cyanoacetate | | 105-34-0 | C4H5NO2 | 200.5 | Di Girolamo et al (2021) | 1923 | |
| 307 | 3-methyl 2-oxazolidinone | | 19836-78-3 | C4H7NO2 | 180/1.5 torr (mp 29) | Di Girolamo et al (2021) | 1952 | |
| 308 | diethanolamine | | 111-42-2 | C4H11NO2 | 268.8 | Driver and Hunter 2020 | 1908 | |
| 309 | ethyl cyanoacetate | | 105-56-6 | C5H7NO2 | 206 | Di Girolamo et al (2021) | 1898 | |
| 310 | 4-formylmorpholine | | 4394-85-8 | C5H9NO2 | 239 | Murray et al 2016 | 1936 | |
| 311 | *N,N*-dimethyllactamide | | 35123-06-9 | C5H11NO2 | 78.6-79.6/4 torr | Clark et al 2017a | 1927 | |
| 312 | nitrobenzene | | 98-95-3 | C6H5NO2 | 210.8 | Murray et al 2016 | 1878 | |
| 313 | 1-(2-hydroxyethyl)-2-pyrrolidone | | 3445-11-2 | C6H11NO2 | 295 | Ho et al 2020 | 1952 | |
| 314 | *N*-(2-methoxy-2-ethoxyethyl)dibutylamine | | 1564250-82-3 | C13H29NO2 | 290.5±15.0 | Samorì et al 2014 | 2014 | |
|  |  | |  |  |  |  |  | |
| 315 | triethanolamine | | 102-71-6 | C6H15NO3 | 335.4 | Diorazio et al 2016 | 1925 | |
| 316 | *N,N*-bis(2-hydroxyethyl)octanamide | | 3077-30-3 | C12H25NO3 | 392.2±27.0 (est) | Moity, Durand et al 2014 | 1957 | |
| 317 | *N,N*-bis(2-hydroxyethyl)decanamide | | 136-26-5 | C14H29NO3 | 417.9±30.0 (est) | Moity, Durand et al 2014 | 1937 | |
|  |  | |  |  |  |  |  | |
| 318 | 1.4-diisocyanatobutane | | 4538-37-8 | C6H8N2O2 | 75-76/14 torr | Di Girolamo et al (2021) | 1932 | |
|  |  | |  |  |  |  |  | |
| 319 | dimethyl sulfide | | 75-18-3 | C2H6S | 37.3 | Driver and Hunter 2020 | 1886 | |
| 320 | thiophene | | 110-02-1 | C4H4S | 84.0 | Diorazio et al 2016 | 1885 | |
| 321 | tetrahydrothiophene | | 110-01-0 | C4H8S | 121.1 | Diorazio et al 2016 | 1910 | |
| 322 | 1-butanethiol | | 109-79-5 | C4H10S | 98.4 | Di Girolamo et al (2021) | 1916 | |
| 323 | diethyl sulfide | | 352-93-2 | C4H10S | 92.1 | Driver and Hunter 2020 | 1879 | |
| 324 | thiane | | 1613-51-0 | C5H10S | 141.8 | Driver and Hunter 2020 | 1910 | |
| 325 | 1.2-ethanedithiol | | 540-63-6 | C2H6S2 | 146 | Di Girolamo et al (2021) | 1905 | |
| 326 | 1,3-dithiolane | | 4829-04-3 | C3H6S2 | 175 | Diorazio et al 2016 | 1928 | |
| 327 | dipropyl disulfide | | 629-19-6 | C6H14S2 | 193.5 | Di Girolamo et al (2021) | 1926 | |
| 328 | dimethyl trithiocarbonate | | 2314-48-9 | C3H6S3 | (mp 219-220) | Di Girolamo et al (2021) | 1931 | |
|  |  | |  |  |  |  |  | |
| 329 | 2-methylthioethanol | | 5271-38-5 | C3H8OS | 81/30 torr | Di Girolamo et al (2021) | 1928 | |
| 330 | diethyl sulfoxide | | 70-29-1 | C4H10OS | 88-89/15 torr | Linke S et al 2020 | 1910 | |
| 331 | tetramethylenesulfoxide | | 1600-44-8 | C4H8OS | 232-234 | Diorazio et al 2016 | 1941 | |
| 332 | *S*-ethyl thioacetate | | 625-60-5 | C4H8OS | 116.4 | Di Girolamo et al (2021) | 1912 | |
| 333 | methyl phenyl sulfoxide | | 1193-82-4 | C7H8OS | 263.5 (mp 32) | Di Girolamo et al (2021) | 1949 | |
| 334 | di-*n*-butyl sulfoxide | | 2168-93-6 | C8H18OS | 105-7/2 torr (mp 32.6) | Di Girolamo et al (2021) | 1924 | |
|  |  | |  |  |  |  |  | |
| 335 | methyl thioglycolate | | 2365-48-2 | C3H6O2S | 148 | Di Girolamo et al (2021) | 1912 | |
| 336 | piperylene sulfone | | 6007-71-2 | C5H8O2S | 85/7 torr | Vinci et al 2007 | 1943 | |
|  |  | |  |  |  |  |  | |
| 337 | dimethylthioformamide | | 758-16-7 | C3H7NS | 96.5-97 | Driver and Hunter 2020 | 1909 | |
| 338 | 1-methyl-2-pyrrolidinethione | | 10441-57-3 | C5H9NS | 144-145/15 torr | Driver and Hunter 2020 | 1955 | |
| 339 | *N,N,N',N*'-tetraethylsulfamide | | 2832-49-7 | C8H20N2O2S | 92/1 torr | Diorazio et al 2016 | 1936 | |
|  |  | |  |  |  |  |  | |
| 340 | perfluoropentane | | 678-26-2 | C5F12 | 29.2 | Wypych and Wypych 2014, 2019 | 1937 | |
| 341 | hexafluorobenzene | | 392-56-3 | C6F6 | 80.2 | Murray et al 2016 | 1947 | |
| 342 | tetradecafluorohexane | | 355-42-0 | C6F14 | 56.6 | Jessop et al 2012 | 1937 | |
| 343 | perfluoro(methylcyclohexane) | | 355-02-2 | C7F14 | 76.3 | Murray et al 2016 | 1946 | |
| 344 | perfluoroheptane | | 335-57-9 | C7F16 | 82.5 | Wypych and Wypych 2014, 2019 | 1943 | |
| 345 | perfluorooctane | | 307-34-6 | C8F18 | 105.9 | Moity, Durand et al 2014 | 1948 | |
| 346 | *cis*-perfluorodecalin | | 60433-11-6 | C10F18 | 142.5 | Diorazio et al 2016 | 1961 | |
| 347 | perfluorodecalin | | 306-94-5 | C10F18 | 142 | Jessop et al 2012 | 1947 | |
| 348 | 1,1,1,3,3-pentafluorobutane | | 406-58-6 | C4H5F5 | 40 | Clark et al 2017a | 1945 | |
| 349 | 1,1,2,2,3,3,4-heptafluoro cyclopentane | | 15290-77-4 | C5H3F7 | 55.9±40.0 (est) | Wypych and Wypych 2014, 2019 | 1966 | |
| 350 | 1,1,1,2,2,4,5,5,5-nonafluoro-4- | | 756-13-8 | C6F12O | 48-49 | Wypych and Wypych 2014, 2019 | 1958 | |
|  | (trifluoromethyl)-3-pentanone | |  |  |  |  |  | |
| 351 | Hexafluoro-*iso*-propanol | | 920-66-1 | C3H2F6O | 59 | Shahbazi et al 2020 | 1961 | |
| 352 | methyl perfluoropropyl ether | | 375-03-1 | C4H3F7O | 33-35 | Wypych and Wypych 2014, 2019 | 1954 | |
| 353 | methyl nonafluorobutyl ether | | 163702-07-6 | C5H3F9O | 64.5 | Wypych and Wypych 2014, 2019 | 1973 | |
| 354 | methyl perfluoro-*iso*-butyl ether | | 163702-08-7 | C5H3F9O | 20.0±40.0 (est) | Wypych and Wypych 2014, 2019 | 1996 | |
| 355 | ethyl perfluorobutyl ether | | 163702-05-4 | C6H5F9O | 76 | Wypych and Wypych 2014, 2019 | 1996 | |
| 356 | ethyl nonafluoro-*iso*-butyl ether | | 163702-06-5 | C6H5F9O | 49.5±40.0 (est) | Wypych and Wypych 2014, 2019 | 1996 | |
| 357 | 1,1,1,2,3,3-hexafluoro-4-(1,1,2,3,3,3- hexafluoropropoxy)pentane | | 870778-34-0 | C8H6F12O | 137.1±40.0 (est) | Wypych and Wypych 2014, 2019 | 2007 | |
| 358 | 3-ethoxy-1,1,1,2,3,4,4,5,5,6,6,6- dodecafluoro-2-(trifluoromethyl)hexane | | 297730-93-9 | C9H5F15O | 126.7±40.0 (est) | Wypych and Wypych 2014, 2019 | 2001 | |
| 359 | 2,3,3,4,4-pentafluorotetrahydro-5-methoxy-2,5-bis[1,2,2,2-tetrafluoro-1-(trifluomethyl)furan | | 957209-18-6 | C11H3F19O2 | 170.2±40.0 (est) | Wypych and Wypych 2014, 2019 | 2007 | |
| 360 | methyl trifluoromethanesulfonate | | 333-27-7 | C2H3F3O3S | 99 | Di Girolamo et al (2021) | 1955 | |
|  |  | |  |  |  |  |  | |
| 361 | dibromomethane | | 74-95-3 | CH2Br2 | 97 | Driver and Hunter 2020 | 1881 | |
| 362 | diodomethane | | 75-11-6 | CH2I2 | 182 | Driver and Hunter 2020 | 1880 | |
| 363 | tetrachloroethlyene | | 127-18-4 | C2Cl4 | 121.3 | Diorazio et al 2016 | 1889 | |
| 364 | trichloroethylene | | 79-01-6 | C2HCl3 | 87.2 | Diorazio et al 2016 | 1906 | |
| 365 | 1,1,2,2-tetrachloroethane | | 70-34-5 | C2H2Cl4 | 146 | Tobiszewski et al 2015 | 1904 | |
| 366 | 1,1-dichloroethane | | 75-34-3 | C2H4Cl2 | 57.4 | Driver and Hunter 2020 | 1880 | |
| 367 | 1,3-dichloropropene | | 142-28-9 | C3H6Cl2 | 120.4 | Tobiszewski et al 2015 | 1921 | |
| 368 | 1-chloropropane | | 540-54-5 | C3H7Cl | 46.6 | Tobiszewski et al 2015 | 1884 | |
| 369 | hexachlorobutadiene | | 87-68-3 | C4Cl6 | 215 | Tobiszewski et al 2015 | 1941 | |
| 370 | 1,4-dichlorobutane | | 110-56-5 | C4H8Cl2 | 161 | Driver and Hunter 2020 | 1907 | |
| 371 | butyl iodide | | 542-69-8 | C4H9I | 130.6 | Driver and Hunter 2020 | 1895 | |
| 372 | 1,3-dichlorobenzene | | 541-73-1 | C6H4Cl2 | 173 | Driver and Hunter 2020 | 1896 | |
| 373 | 1,4-dichlorobenzene | | 106-46-7 | C6H4Cl2 | 174 | Driver and Hunter 2020 | 1895 | |
| 374 | 3-chlorophenol | | 108-43-0 | C6H5ClO | 214 | Driver and Hunter 2020 | 1908 | |
| 375 | bromobenzene | | 108-86-1 | C6H5Br | 156.2 | Diorazio et al 2016 | 1862 | |
| 376 | iodobenzene | | 591-50-4 | C6H5I | 188.4 | Diorazio et al 2016 | 1889 | |
|  |  | |  |  |  |  |  | |
| 377 | 2-chloroethanol | | 107-07-3 | C2H5ClO | 128-130 | Driver and Hunter 2020 | 1877 | |
| 378 | ethyl chloroacetate | | 105-39-5 | C4H7ClO2 | 144-146 | Driver and Hunter 2020 | 1882 | |
| 379 | methyl trichloroacetate | | 598-99-2 | C3H3Cl3O2 | 154 | Di Girolamo et al (2021) | 1954 | |
| 380 | ethyl trichloroacetate | | 515-84-4 | C4H5Cl3O2 | 167.5 | Driver and Hunter 2020 | 1917 | |
| 381 | bis(2-chloroethyl)ether | | 111-44-4 | C4H8Cl2O | 178.5 | Driver and Hunter 2020 | 1921 | |
| 382 | benzoyl chloride | | 98-88-4 | C7H5ClO | 197.2 | Driver and Hunter 2020 | 1879 | |
| 383 | benzoyl bromide | | 618-32-6 | C7H5BrO | 218.5 | Driver and Hunter 2020 | 1903 | |
|  |  | |  |  |  |  |  | |
| 384 | tetramethylsilane | | 75-76-3 | C4H12Si | 26.6 | Wypych and Wypych 2014, 2019 | 1911 | |
| 385 | hexamethyldisiloxane | | 107-46-0 | C6H18OSi2 | 99.5 | Clark et al 2017a | 1944 | |
| 386 | decamethylcyclopentasiloxane | | 541-02-6 | C10H30O5Si5 | 210 | Moity, Durand et al 2014 | 1945 | |
|  |  | |  |  |  |  |  | |
|  | **BLENDS/MIXTURES** | |  |  |  |  |  | |
| 387 | oxymethylene dimethyl ethers | | 24991-52-4 |  |  | Zhenova et al 2019 | 1961 | |
| 388 | ABE acetone, butanol, ethanol | |  |  |  | Bankar et al 2013 |  | |
| 389 | lignin pyrolysis oil methyl ether | |  |  |  | Mudraboyina et al 2016 |  | |
| 390 | soya bean oil methyl ester methyl soyate | | 67784-80-9 |  |  | Clark et al 2017a |  | |
| 391 | methyl 5-(dimethylamino-2-methyl-5-oxopentanoate | | 1174627-68-9 | C9H17NO3 | 278-282 | Cseri and Szekely 2019 | 2009 | |
| 392 | tetraethylene glycol | | 112-60-7 | C8H18O5 | 327 | Moity, Durand et al 2014 | 1932 | |
| 393 | polyethylene glycol PEG-400 | | 25322-68-3 |  | >325 (mp 57.5) | Shakeel et al 2014 | 1929 | |
| 394 | polyethylene glycol PEG-600 | | 25322-68-3 |  | >325 (mp 57.5) | Calvo‑Flores et al 2018 | 1929 | |
| 395 | polypropylene glycol PPG-1200 | | 25322-69-4 |  | >300 (mp 50-70) | Calvo‑Flores et al 2018 | 1944 | |
| 396 | dipropylene glycol dimethyl ether | | 89399-28-0 | C8H18O3 | 178.8±15.0 | Murray et al 2016 | 1985 | |
|  | (mixture of 396 and 397) | |  |  |  |  |  | |
| 397 | 2,2'-oxybis(1-methoxypropane) | | 189354-80-1 | C8H18O3 | 178.8±15.0 | Murray et al 2016 | 2010 | |
| 398 | 1,1'-oxybis(2-methoxypropane) | | 63019-84-1 | C8H18O3 | 178.8±15.0 | Murray et al 2016 | 2004 | |
| 399 | glycerol formal (mixture of 399 and 400) | | 5464-28-8 | C4H8O3 |  | Jessop et al 2012 | 1928 | |
| 400 | 1,3-dioxan-5-ol (55% of glycerol formal) | | 4740-78-7 | C4H8O3 | 193.8 | Clark et al 2017a | 1928 | |
| 401 | 1,3-dioxolan-4-yl methanol (45% of glycerol formal) | | 5464-28-8 | C4H8O3 | 84-85/11 torr | Clark et al 2017a | 1928 | |
| 402 | turpentine oil | | 8006-64-2 |  |  | Clark et al 2017a |  | |
| 403 | polydimethylsiloxane | | 63148-62-9 |  |  | Calvo‑Flores et al 2018 | 1961 | |
| 404 | mixture of dimethyl succinate, glutarate and adipate | | 95481-62-2 |  |  | Wypych and Wypych 2014, 2019 | 1985 | |
|  |  | |  |  |  |  |  | |
|  | **OTHERS** | |  |  |  |  |  | |
| 405 | sucrose acetate hexaisobutyrate | | 126-13-6 | C40H62O19 |  | Wypych and Wypych 2014, 2019 | 1960 | |
| 406 | cholinium acetate | | 14586-35-7 | C7H18NO3 | (mp 51) | Moity, Durand et al 2014 | 1966 | |
| 407 | 1-ethyl-3-methylimidazolium bis(trifluoromethylsulfonyl) imide | | 174899-82-2 | C8H11F6N3O4S2 | 543.6 | Wypych and Wypych 2014, 2019 | 1996 | |
| 408 | 1-ethyl-3-methylimidazolium bis(fluorosulfonyl)imide | | 235789-75-0 | C6H11F2N3O4S2 |  | Wypych and Wypych 2014, 2019 | 1999 | |
| 409 | 3-butyl-1-methylimidazolium tetrafluoroborate | | 174501-65-6 | C8H15BF4N2 |  | Moity, Durand et al 2014 | 1996 | |
| 410 | tributyltetradecylphosphonium chloride | | 81741-28-8 | C26H56ClP |  | Wypych and Wypych 2014, 2019 | 1962 | |
| 411 | tetradecyltrihexylphosphonium chloride | | 258864-54-9 | C32H68ClP |  | Wypych and Wypych 2014, 2019 | 2001 | |
|  |  | |  |  |  |  |  | |
|  | **INORGANIC including ESTERS** | |  |  |  |  |  | |
| 412 | carbon dioxide | | 124-38-9 | CO2 |  | Jessop et al 2012 |  | |
| 413 | hydrazine | | 302-01-2 | H4N2 | 113.5 | Driver and Hunter 2020 | 1888 | |
|  |  | |  |  |  |  |  | |
| 414 | trimethyl borate | | 121-43-7 | C3H9BO3 | 67-68 | Di Girolamo et al (2021) | 1931 | |
|  |  | |  |  |  |  |  | |
| 415 | phosphoryl chloride | | 10025-87-3 | Cl3OP | 105.8 | Driver and Hunter 2020 | 1880 | |
| 416 | trimethyl phosphate | | 512-56-1 | C3H9O4P | 85 | Driver and Hunter 2020 | 1926 | |
| 417 | triethylphosphate | | 78-40-0 | C6H15O4P | 215-216 | Driver and Hunter 2020 | 1915 | |
| 418 | tributyl phosphate | | 126-73-8 | C12H27O4P | 177-178/27 torr | Driver and Hunter 2020 | 1928 | |
|  |  | |  |  |  |  |  | |
| 419 | thionyl chloride | | 7719-09-7 | Cl2OS | 76 | Driver and Hunter 2020 | 1880 | |
| 420 | sulfuric acid | | 7664-93-9 | H2O4S | ~290 | Driver and Hunter 2020 | 1878 | |
| 421 | ethylene sulfite | | 3741-38-6 | C2H4O3S | 173 | Diorazio et al 2016 | 1926 | |
| 422 | dimethyl sulfite | | 616-42-2 | C2H6O3S | 126 | Diorazio et al 2016 | 1911 | |
| 423 | diethyl sulfite | | 623-81-4 | C4H10O3S | 158 | Wypych and Wypych 2014, 2019 | 1885 | |
| 424 | dimethyl sulfate | | 77-78-1 | C2H6O4S | 187.5 | Driver and Hunter 2020 | 1868 | |
| 425 | diethyl sulfate | | 64-67-5 | C4H10O4S | 208 | Driver and Hunter 2020 | 1879 | |

**References**

Bankar SB, Survase SA, Ojamo H and Granstöm T (2013) Biobutanol: the outlook of an academic and industrialist. RSC Adv **3:**24734-24757

Barbaro P, Liguori F, Oldani C and Moreno-Marrodán C (2020) Sustainable Catalytic Synthesis for a Bio-Based Alternative to the Reach-Restricted N-Methyl-2-Pyrrolidone. Adv Sustainable Syst **4:**1900117

Bauer MlC and Kruse A (2019) The use of dimethyl ether as an organic extraction solvent for biomass applications in future biorefineries: A user-oriented review. Fuel **254:**115703

Bergez-Lacoste M, Thiebaud-Roux S, De Caro P, Fabre JF, Gerbaud V and Mouloungui Z (2014) From chemical platform molecules to new biosolvents: design engineering as a substitution methodology. Biofuels Bioprod Bioref **8:**438-451

Byrne FP, Forier B, Bossaert G, C Hoebers C, Farmer TJ and Hunt AJ (2018) A methodical selection process for the development of ketones and esters as bio-based replacements for traditional hydrocarbon solvents. Green Chem **20**:4003

Calvo‑Flores FG, Monteagudo‑Arrebola MJ, Dobado JA and Isac‑García J (2018) Green and Bio-Based Solvents. Top Curr Chem (Z) **376:**18

Christy S, Noschese A, Lomelí-Rodriguez M, Greeves N and Lopez-Sanchez JA (2018) Recent progress in the synthesis and applications of glycerol carbonate. Curr Opin Green Sustain Chem 14:99–107

Clark JH, Hunt A, Topi C, Paggiola G and Sherwood J (2017a) An Appendix of Solvent Data Sheets. Sustainable Solvents: Perspectives from Research, Business and International Policy (Green Chemistry Series No. 49) Ch 6 pp235-347 Royal Society of Chemistry

Cseri L and Szekely G (2019) Towards cleaner PolarClean: efficient synthesis and extended applications of the polar aprotic solvent methyl 5-(dimethylamino)-2-methyl-5-oxopentanoate. Green Chem **21:**4178-4188

Di Girolamo D, Pascual J, Aldamasy MH, Iqbal Z, Li G, Radicchi E, Li M, Turren-Cruz S-H, Nasti G, Dallmann A, De Angelis F and Abate A (2021) Solvents for Processing Stable Tin Halide Perovskites ACS Energy Lett 6:959−968

Diorazio LJ, Hose DRJ and Adlington NK (2016) Towards a More Holistic Framework for Solvent Selection. Org Process Res Dev **20:**760-773

Driver MD and Hunter CA (2020) Solvent similarity index. Phys Chem Chem Phys **22:**11967-11975

Estévez C (2009) Sustainable Solutions – Green Solvents for Chemistry. Sustainable Solutions for Modern Economies (RSC Green Chemistry No. 4) Höfer R (ed) Ch10 pp407-424 Royal Society of Chemistry

Gevorgyan A, Hopmann KH and Bayer A (2020) Exploration of New Biomass-Derived Solvents: Applications to Carboxylation Reactions. ChemSusChem **13:**2080-2088

Ho D, Lee J, Park S, Park Y, Cho K, Campana F, Lanari D, Facchetti A, Seo SY, Kim C, Marrocchi A and Vaccaro L (2020) Green solvents for organic thin-film transistor Processing. J Mater Chem C **8:**5786-5794

Jessop PG, Jessop DA, Fu D and Phan L (2012) Solvatochromic parameters for solvents of interest in green chemistry. Green Chem **14:**1245-1259

Jin S, Byrne F, McElroy CR, Sherwood J, Clark JH and Hunt AJ (2017) Challenges in the development of bio-based solvents: a case study on methyl(2,2-dimethyl-1,3-dioxolan-4-yl)methyl carbonate as an alternative aprotic solvent. Faraday Discuss **202:**157-173

Kobayashi S, Tamura T, Yoshimoto S, Kawakami T and Masuyama A (2019) 4-Methyltetrahydropyran (4-MeTHP): Application as an Organic Solvent. Chem Asian J **14:**3921-3937

Lee J, Byranvand MM, Kang G, Son SY, Song S, Kim G.-W. and Park T (2017) Green-Solvent-Processable, Dopant-Free Hole-Transporting Materials for Robust and Efficient Perovskite Solar Cells. J Am Chem Soc 139:12175−12181

Linke S, McBride K and Sundmacher K (2020) Systematic Green Solvent Selection for the Hydroformylation of Long-Chain Alkenes. ACS Sustainable Chem Eng **8:**10795-10811

Marcel R, Durillon T, Djakovitch L, Fache F and Rataboul F (2019) First Example of the Use of Biosourced Alkyl Levulinates as Solvents for Synthetic Chemistry: Application to the Heterogeneously Catalyzed Heck Coupling. ChemistrySelect **4:**3329-3333

Moity L, Durand M, Benazzouz A, Molinier V and Aubry J-M (2014) In Silico Search for Alternative Green Solvents. Chemat F and Abert Vian M (eds) Alternative Solvents for Natural Products Extraction, Green Chemistry and Sustainable Technology Ch 1pp3-24, Springer-Verlag, Berlin Heidelberg

Moity L. Molinier V, Benazzouz A, Barone R. Marion P and Aubry J-M (2014) In silico design of bio-based commodity chemicals: application to itaconic acid based solvents. Green Chem. **16:**146-160

Mudraboyina BP, Farag S, Banerjee A, Chaouki J and Jessop PG (2016) Supercritical fluid rectification of lignin pyrolysis oil methyl ether (LOME) and its use as a bio-derived aprotic solvent. Green Chem **18:**2089-2094

Murray PM, Bellany F, Benhamou L, Bučar D-K, Tabor AB and Sheppard TD (2016) The application of design of experiments (DoE) reaction optimisation and solvent selection in the development of new synthetic chemistry. Org Biomol Chem **14:**2373-2384

Pellis A, Byrne FP, Sherwood J, Vastano M, Comerford JW and Farmer TJ (2019) Safer bio-based solvents to replace toluene and tetrahydrofuran for the biocatalyzed synthesis of polyesters. Green Chem **21:**1686-1694

Qian S, Liu XY, Emel’yanenko VN, Sikorski P, Kammakakam I, Flowers, BS, Jones TA, Turner CH, Verevkin, SP and Bara JE (2021) Synthesis and Properties of 1,2,3-Triethoxypropane: A Glycerol Derived Green Solvent Candidate. Ind Eng Chem Res **59**:20190-20200

Samorì C, Pezzolesi L, Barreiro DL, Galletti P, Pasteris A and Tagliavini E (2014) Synthesis of new polyethoxylated tertiary amines and their use as Switchable Hydrophilicity Solvents. RSC Adv **4:**5999-6008

Shahbazi S, Li M-Y, Fathi A and Diau EW-G (2020) Realizing a Cosolvent System for Stable Tin-Based Perovskite Solar Cells Using a Two-Step Deposition Approach. ACS Energy Lett 5:2508-2511

Shakeel F, Haq N, Alanani FK and Alsarra IA (2014) Measurement and Correlation of Solubility of Olmesartan Medoxomil in Six Green Solvents at 295.15-330.15 K. Ind Eng Chem Res **53:**2846-2849

Sherwood J, Parker HL, Moonen K, Farmer TJ and Hunt AJ (2016) N-Butylpyrrolidinone as a dipolar aprotic solvent for organic synthesis. Green Chem **18:**3990-3996

Strohmann M, Bordet A, Vorholt AJ and Leitner W (2019) Tailor-made biofuel 2-butyltetrahydrofuran from the continuous flow hydrogenation and deoxygenation of furfuralacetone. Green Chem **21:**6299-6306

Tobiszewski M, Tsakovski S, Simeonov V, Namieśnika J and Pena-Pereira F (2015) A solvent selection guide based on chemometrics and multicriteria decision analysis. Green Chem **17:**4773-4785 (and correction: Green Chem 17:5206)

Vinci D, Donaldson M, Hallett JP, John EA, Pollet P, Thomas CA, Grilly JD, Jessop PG, Liotta CL and Eckert CA (2007) Piperylene sulfone: a labile and recyclable DMSO substitute. Chem Commun 1427-1429

Wypych A and Wypych G (2014) Databook of Green Solvents. ChemTech Publishing Toronto

Wypych A and Wypych G (2019) Databook of Green Solvents 2^nd^ edn. ChemTech Publishing Toronto

Yang W and Sen A (2010) One-Step Catalytic Transformation of Carbohydrates and Cellulosic Biomass to 2,5-Dimethyltetrahydrofuran for Liquid Fuels. ChemSusChem **3**:597

Yara-Varón E, Selka A, Fabiano-Tixier AS, Balcells M, Canela-Garayoa R, Bily A, Touaibiac M and Chemat F (2016) Solvent from forestry biomass. Pinane a stable terpene derived from pine tree byproducts to substitute n-hexane for the extraction of bioactive compounds. Green Chem 18:6596

Zhenova A, Pellis A, Milescu RA, McElroy CR, White RJ and Clark JH (2019) Solvent Applications of Short-Chain Oxymethylene Dimethyl Ether Oligomers. ACS Sustainable Chem Eng **7:**14834-14840
